# Characterization of single-nucleotide variation in Indian-origin rhesus macaques (*Macaca mulatta*)

**DOI:** 10.1186/1471-2164-12-311

**Published:** 2011-06-13

**Authors:** Gloria L Fawcett, Muthuswamy Raveendran, David Rio Deiros, David Chen, Fuli Yu, Ronald Alan Harris, Yanru Ren, Donna M Muzny, Jeffrey G Reid, David A Wheeler, Kimberly C Worley, Steven E Shelton, Ned H Kalin, Aleksandar Milosavljevic, Richard Gibbs, Jeffrey Rogers

**Affiliations:** 1 Human Genome Sequencing Center, Department of Molecular and Human Genetics, Baylor College of Medicine, Houston, Texas 77030, USA; 2 Department of Molecular and Human Genetics, Baylor College of Medicine, Houston, Texas 77030, USA; 3 Southwest National Primate Research Center, San Antonio, Texas 78245, USA; 4 Department of Psychiatry, the HealthEmotions Research Institution, University of Wisconsin-Madison, Madison, Wisconsin, 53719, USA; 5 Department of Psychology, Waisman Laboratory for Brain Imaging and Behavior, University of Wisconsin-Madison, Madison, Wisconsin, 53719, USA

**Keywords:** single nucleotide polymorphism, common variants, SOLiD™, genetic variation, rhesus macaque

## Abstract

**Background:**

Rhesus macaques are the most widely utilized nonhuman primate model in biomedical research. Previous efforts have validated fewer than 900 single nucleotide polymorphisms (SNPs) in this species, which limits opportunities for genetic studies related to health and disease. Extensive information about SNPs and other genetic variation in rhesus macaques would facilitate valuable genetic analyses, as well as provide markers for genome-wide linkage analysis and the genetic management of captive breeding colonies.

**Results:**

We used the available rhesus macaque draft genome sequence, new sequence data from unrelated individuals and existing published sequence data to create a genome-wide SNP resource for Indian-origin rhesus monkeys. The original reference animal and two additional Indian-origin individuals were resequenced to low coverage using SOLiD™ sequencing. We then used three strategies to validate SNPs: comparison of potential SNPs found in the same individual using two different sequencing chemistries, and comparison of potential SNPs in different individuals identified with either the same or different sequencing chemistries. Our approach validated approximately 3 million SNPs distributed across the genome. Preliminary analysis of SNP annotations suggests that a substantial number of these macaque SNPs may have functional effects. More than 700 non-synonymous SNPs were scored by Polyphen-2 as either possibly or probably damaging to protein function and these variants now constitute potential models for studying functional genetic variation relevant to human physiology and disease.

**Conclusions:**

Resequencing of a small number of animals identified greater than 3 million SNPs. This provides a significant new information resource for rhesus macaques, an important research animal. The data also suggests that overall genetic variation is high in this species. We identified many potentially damaging non-synonymous coding SNPs, providing new opportunities to identify rhesus models for human disease.

## Background

Experimental study of rhesus macaques (*Macaca mulatta*) is critical to progress in many aspects of biomedical research. This species is widely used in neurobiology and behavioral science [1-5], endocrinology [6,7], physiological studies of cardiovascular disease and obesity [8], diabetes [9], alcoholism and addiction research [10,11], and other diverse fields [12,13]. Rhesus macaques are central to progress in the development of vaccines and anti-viral therapies against HIV infection and AIDS because these animals are the best available animal model for studies of lentivirus infection [14,15]. However, despite the wide use of rhesus macaques in research, this species is under-utilized as a model organism for investigation of genetic processes influencing human biology and human disease. One factor limiting the use of rhesus monkeys in genetic analyses is the lack of existing information about intra-species polymorphism. Currently, dbSNP contains fewer than 900 validated SNPs for this species (http://www.ncbi.nlm.nih.gov/projects/SNP/) [16] and only a small number of copy number variants (CNVs) have been described [17,18]. Clearly, more information about genetic variation segregating within this otherwise widely studied species would be useful.

Additional effort to identify both common and low frequency genetic variation in this species is warranted, based on the potential opportunity for and significance of developing new genetic models of human disease. Despite the limited information available today, a number of studies have shown that rhesus monkeys exhibit intra-species genetic variation in the same genes that have been associated with risk of disease in humans. Furthermore, recent analyses have demonstrated that DNA sequence variation among rhesus macaques can parallel the functional effects of known variation in humans, providing remarkably similar and thus highly informative animal models of human genetic risk. In one example, researchers found natural mutations segregating among captive rhesus monkeys in two genes that had previously been associated with macular degeneration in humans [19]. The investigators found that, as in humans, the polymorphisms in macaques were associated with individual variation in the major risk factor for macular degeneration, demonstrating a parallel genetic effect in rhesus and establishing this as an important model of genetic predisposition to this disease. Other examples of genetic variation among rhesus macaques that parallels human functional variation are known in the serotonin transporter [20,21] and the mu-opioid receptor [22].

Based on these observations, we anticipated that genome-wide knowledge of SNP variation segregating within research colonies of Indian-origin rhesus macaques would produce novel and valuable information about functionally significant genetic variation in a number of important cellular pathways. Broad knowledge of both common and low frequency sequence variation in rhesus macaques (and by implication, equivalent information for other species of nonhuman primates) will facilitate discovery of new animal models of functional variation in specific genetic pathways and thus help investigators to elucidate mechanisms associated with human disease [17]. It is important to recognize that nonhuman primates constitute a unique set of animal models that offer a singular combination of research advantages. The overall genetic and physiological similarity of macaques and other nonhuman primates to humans [3,23] coupled with the opportunity to examine sets of animals raised under controlled environmental circumstances, or subject to precise environmental manipulations (e.g. exposure to therapeutic drugs or changes in diet), creates outstanding research opportunities. Analyses in macaques can examine the specific effects of individual genetic variants on those cellular pathways, the effects of genotype-by-genotype or genotype-by-environment interaction, or other complex processes that are relevant to human disease but cannot be readily examined in human subjects under such tightly controlled environments.

Unfortunately, little is known about genetic variation in rhesus macaques. Fewer than 900 validated SNPs and 100 CNVs have been described. The available data [23-28] suggest that a given number of rhesus macaques will show as much or more diversity and heterozygosity as the same number of humans. We undertook this study to assess the value of whole genome re-sequencing in rhesus macaques for the discovery of novel SNPs. We identified and bioinformatically validated SNPs throughout the rhesus macaque genome using existing sequence data and new SOLiD sequencing reads we generated from three unrelated macaques. We are particularly interested in the identification of novel variation among these animals that has potentially significant effects on gene function and physiological processes relevant to human disease.

## Results

**Figure 1:**
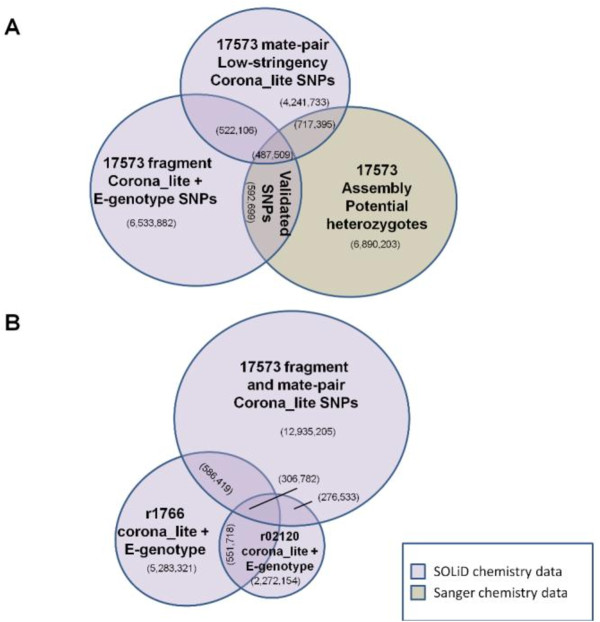
**Validation by category, direct comparison examples**. Only the largest data sets are shown. (A) Validation in a single animal with multiple chemistries. All data for the reference animal 17573 are displayed by chemistry. SNPs falling into the overlap region are considered to be validated. (B) Validation in multiple animals with a single chemistry and with multiple chemistries. SOLiD data from re-sequencing efforts were compared to the unrelated animals r1766 and r02120. A large number (603,463) of additional SNPs not identified in the reference animal were identified and validated by comparing r02120and r1766. We observed roughly one-third the number of validated SNPs from r02120 that were obtained using the sequence data from r1766, due to reduced uniquely mapping sequence coverage in r02120 (Table 1).

We identified and validated more than 3 million unique SNPs in rhesus macaques through pairwise comparisons of SNP data sets (Table 1). Our starting point for these analyses was a list of 4.3 million inferred heterozygous positions in the original Sanger sequencing data for the reference animal [23]. Comparison of these Sanger data with corona_lite SNP calls from SOLiD re-sequencing of the reference animal resulted in validation of 1,056,266 heterozygous positions (Figure 1). Similarly, re-sequencing of two additional Indian-origin animals, followed by comparison to the reference animal SNP list, resulted in the validation of an additional 873,552 SNPs.

**Table 1:** Bioinformatically validated SNPs

	17573 Sanger	17573 SOLiD	r1766	r02120	Sub-species comparison	MamuSNP	ENCODE	dbSNP
17573 Sanger								
	
17573 SOLiD	1,056,266							
	
r1766	22,665	412,920						
	
r02120	5,734	103,323	328,910					
	
Sub-species comparison	4,185	6,519	1,070	226				
	
MamuSNP	389	781	159	33	19			
	
ENCODE	29	76	14	7	0	1		
	
dbSNP	11	12	5	0	0	0	0	
	
egeno.assembly.hets	--	421,234	64,584	41,607	34,339	--	--	--
	
egeno.17573.SOLiD	--	--	109,485	61,941	9	--	--	--
	
egeno.r1766.frag	--	155,454	--	93,016	17	--	--	--
	
egeno.r02120.frag	--	41,836	69,424	--	3	--	--	--
	
egeno.sub-species comp	--	677	743	443	--	--	--	--

Unique non-overlapping validated SNPs identified in each pair-wise comparison are displayed. Validated SNPs were determined on the basis of both alleles at a genomic location being identified in at least two data sets and one of either multiple animals or multiple chemistries. Validated SNPs that fit into multiple cells were represented in the left-most appropriate cell.

We were able to validate 1,058,581 additional SNPs among just three rhesus macaques using e-genotyping (http://is04607.com/~drio/egenotype/[29]). E-genotyping is a novel *in silico *method to survey mapped read sequence data using 31bp probe sequences created from a list of known or suspected SNPs. Unvalidated potential heterozygous positions from the original Sanger reference animal data were used to create uniquely mapping e-genotyping probes. Using these probes we screened each of the SOLiD read sets (i.e. the reference animal and the two additional animals). This identified 527,425 validated SNPs that were not validated using the Sanger data and corona_lite analyses of SOLiD data. Probes created from the corona_lite analyses of SOLiD data were used to identify and validate 531,156 more SNPs.

Finally, we used the smaller published datasets to complete a comprehensive list of validated SNPs. We validated 49,747 SNPs by comparing the results from the corona_lite re-sequencing analyses with previously published datasets. A small number of SNPs (n = 20) were validated through comparisons among the previously published data. These 20 SNPs were not found in our new SOLiD re-sequencing data (Figure S1, Additional file 1).

**Figure 2:**
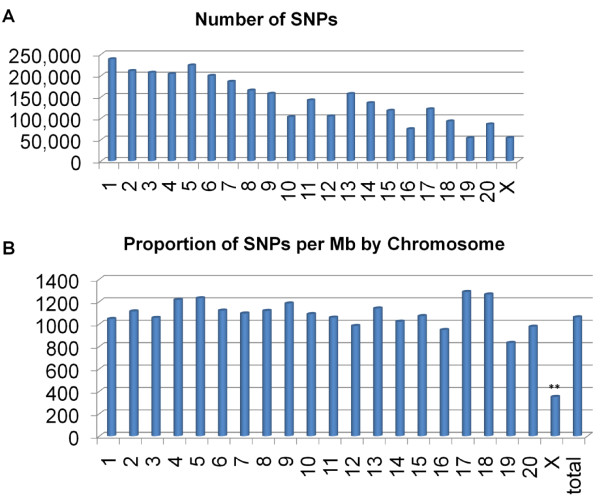
**Genomic distribution of SNPs**. (A) The total number of SNPs identified in each chromosome. (B) The second bar graph shows the relative concentrations of SNPs validated in SNPs/Mb by chromosome. Only chromosome X (**) displays a SNP concentration that deviates more than one standard deviation unit (195.3 SNPs/Mb) from the mean (1057.1 SNPs/Mb).

The genomic distribution of the validated SNPs appears to be random across the autosomes (Figure 2). SNP distribution by chromosome was consistent with chromosome length, except for the X chromosome. X chromosome SNP discovery is limited in this study because one of the three study animals was male (r02120). Across the autosomes, the density of SNPs per Mb fits within one standard deviation (195.3 SNPs/Mb) from the overall means (1,057.1 SNPs/Mb). We retained lists of the unvalidated SNPs in order to facilitate future efforts at SNP discovery in genomic/genic regions where coverage is relatively sparse. This data is provided in our Genboree website (http://genboree.org/java-bin/project.jsp?projectName=Rhesus%20SNPs%20using%20Next-Gen%20Sequencing&isPublic=Yes[30]) in the "Rhesus SNPs" database in a track called "Unvalidated:SNPs".

Unidentified nearly identical duplications of sequence (e.g. unrecognized segmental duplications) in the rhesus whole genome assembly are one potential cause of false positive SNPs. If nearly identical duplications were mistakenly collapsed in the genome assembly, then wherever there are single base differences between two copies of a sequence, the reference animal would appear heterozygous for a SNP. The other two animals would also appear heterozygous for the same SNP, thus providing false validation. We addressed this issue in several ways. First, we note that this problem would produce false heterozygote calls across all three animals, and we found that only ~10% of all of our validated SNPs are scored as heterozygous in all three animals (note however, that the animal with the lowest coverage, r02120, limits the number of SNPs identified as heterozygous in all sequenced animals). Among the 306,782 SNPs that are heterozygous in all subjects, we can evaluate the impact of cryptic sequence duplication in two ways. We compared Sanger read coverage for these 306,782 SNPs against an equivalent number of SNPs that were scored as heterozygous in two animals and homozygous in one animal. Unrecognized duplications should have higher than average read coverage in the original 5.2 × Sanger whole genome read data. We found (Figure S2, Additional file 2) that the mean coverage and read coverage distributions for the two sets of SNPs is virtually identical. If a substantial fraction of the "three heterozygote" SNPs were false positives due to unrecognized duplications in the genome assembly, we would expect a higher mean read coverage for that set compared with the control SNPs that are not scored as heterozygous in all three animals. The percentage of SNPs with read coverage of 11 or greater (i.e. twice the genome-wide average) was only 3.8% for SNPs detected in all three individuals and 3.5% for SNPs detected in only two individuals (0.38% and 0.35% respectively of all of our validated SNPs).

An alternative hypothesis is that duplicated sequences within the rhesus assembly were not assembled in the chromosome scaffolds and were instead placed in the "chromosome unknown" or chrUr bin. The tendency for duplications to be included in unknown chromosomes has been noted in other whole genome shotgun assemblies [31]. Therefore we wished to determine whether our validated SNPs had homology to assembled contigs placed in chrUr, which might represent unrecognized duplications. To test this, we split chrUr into 150 bp length "reads" and scanned this simulated "read" data using e-genotype probes created from all of our validated rhesus SNPs (3,271,622 probes, representing the reference position at all locations as well as the one or two non-reference validated alleles). None of the probes matched sequence from the unmapped reference sequence provided in chrUr. Given these various results, we conclude that there is no clear evidence for a significant number of false positive SNP calls due to duplicated genomic regions in our SNP data.

### SNP annotation and Polyphen Analysis

**Figure 3:**
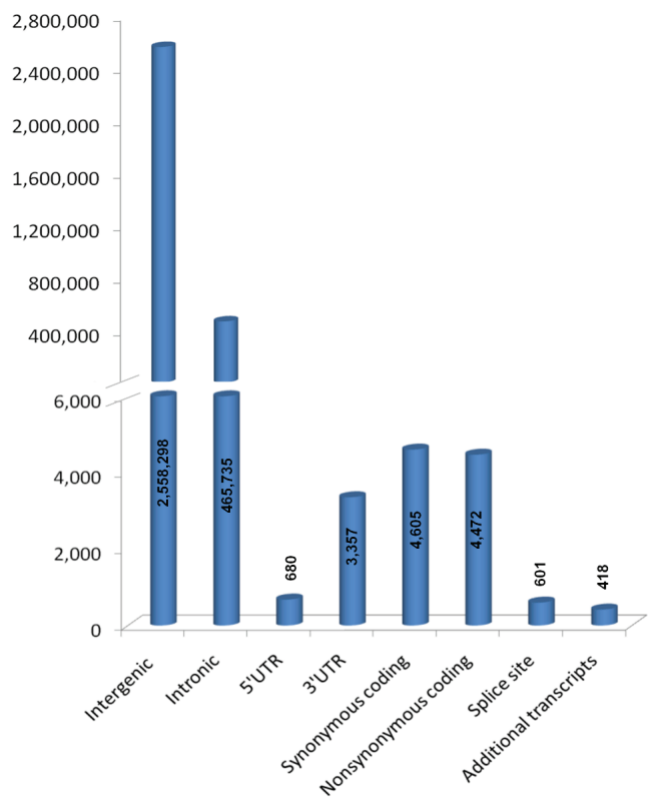
**SNP annotation**. Genomic context of annotated validated SNPs. All annotations were obtained from Ensemble build 57 (released March 2010). The number of SNPs that fit into each category represented by a bar is listed over the respective bar. The bar for "Additional transcripts" represents only those SNPs where the specific annotation placement information was different between different transcripts for the same SNP.

All the validated SNPs were further examined to characterize their position relative to known coding regions and possible functionality (Figure 3). Due to the incomplete annotation of the rhesus reference genome, the annotations of rhesus SNPs are based in part on mapping to homologous human transcripts. As expected, most validated SNPs map to intergenic regions (84.2% of validated SNPs). Intronic SNPs were the second most prevalent at 15.3%. Very few (601 or 0.02%) of the SNPs fell into known or predicted splice sites. Among coding SNPs, about equal numbers are synonymous changes (4,605, 0.15% of total) and non-synonymous (4,472, 0.15% of total). A number of SNPs fell into regions of alternative splicing such that the genomic placement of the SNP varied by transcript. All such SNPs (n = 418) are associated with a coding sequence of at least one transcript, and with an alternative transcript where the placement differed: synonymous coding (21.5%), non-synonymous coding (19.1%), 5'UTR (28.9%), 3'UTR (30.4%).

Potentially functional polymorphisms in rhesus macaques are of particular interest, especially those in genes that may correlate with human disease. The rhesus genome annotation is incomplete and based primarily upon gene prediction algorithms that rely upon human-to-rhesus homology [32-34]. Therefore, we relied upon a homology-based method (see Methods) to generate hypotheses about the functionality of non-synonymous SNPs (nsSNPs) (Table 2). A total of 4,439 SNPs from the full list of nsSNPs (4,472) were successfully converted from rheMac2 coordinates to hg18 coordinates. After removing all SNPs where the hg18 reference allele matched the new variant macaque allele, 4,177 nsSNPs were submitted to Polyphen-2 (http://genetics.bwh.harvard.edu/pph2/[35]) [36]. Of those, 411 were scored as probably damaging and 325 scored as possibly damaging. SNPs that affect binding sites or are located in transmembrane regions can often be expected to alter protein function. Twelve of the probably damaging mutations are annotated as falling in transmembrane regions, two affect a metal binding site, and one affects a modified residue. Of the possibly damaging mutations, six are in transmembrane regions, one is in a propeptide, two affect modified residues, and one affects a carbohydrate binding site. We performed GeneGo (http://www.genego.com/[37]) pathway analysis to determine which diseases were associated with the genes containing predicted deleterious rhesus SNPs from the PolyPhen-2 analysis. These genes were involved in one or more critical organism process such as apoptotic pathways, DNA repair, development or inflammation. Table 2 displays a non-exhaustive list of diseases associated with the relevant pathways, many of which are due to a few well-known genes: *Brca2*, *Ppgca1b*, *Map3k1*, and *Casp7*.

**Table 2:** Diseases associated with genes containing nsSNPs

General Category	# Associated Deleterious SNPs	Gene names	Diseases		
Apoptosis	8	*CYCS;*	Huntington's disease	DNA damage	Alzheimer's disease
		*TNFRSF10D;*	Thrombocytopenia	Bipolar	Rheumatoid arthritis
		*CASP7; MAP3K1;*	Diabetes mellitus type I	Renal cell carcinoma	Myeloproliferative disorders
		*PRKCZ; MPL;*			
		*TNFRSF19*	Leukemia	Diabetes mellitus type II	Amyotrophic lateral Sclerosis
			Pancreatitis	Carcinoma (multiple types)	Multiple sclerosis

Cell cycle	20	*BRCA2; POLD2;*	Spontaneous abortion	Genomic instability	Drug toxicity
		*CDC14A; CDT1;*			
		*CHAF1A; WRN;*	Endometriosis	Anemia	Li-Fraumeni Syndrome
		*IPO5; KIF23; TTK;*	Aneuploidy	Amyotrophic lateral Sclerosis	Diabetes mellitus type I
		*MSH2; CASC5;*			
		*PRKCZ; CASP7;*	Carcinoma (multiple types)	Diabetes mellitus type II	Werner Syndrome
		*SERPINB13;*			
		*LATS1*	Male infertility	Rheumatoid arthritis	osteoporosis

Development	11	*NCOA6; PABPN1;*	Carcinoma (multiple types)	Endometriosis	Asthma
		*LTF; MAP3K1;*			
		*TLL1; YAP1; SPEN;*	Deglutition disorders	Male infertility	Coronary disease
		*IL18R1*	Oculopharyngeal Muscular dystrophy	Li-Fraumeni Syndrome	Idiopathic pulmonary fibrosis

DNA repair	9	*BRCA2; CHAF1A;*	Genomic instability	Werner Syndrome	Fanconi anemia
		*FANCM; RAD51L1;*	Carcinoma (multiple types)	Diabetes mellitus type I	Drug toxicity
		*WRN; MSH2*			

Inflammation	22	*CYCS; SOCS6;*	Huntington's disease	Severe combined Immunodeficiency	Stomach ulcer
		*BDKRB1; HPRT1;*			
		*IL18R1; IL7R;*	Thrombocytopenia	Multiple sclerosis	Ataxia
		*MAP3K1; 1L27RA;*	Asthma	Rheumatoid arthritis	Leukemia
		*PRKCZ; OAS2;*	Diabetes mellitus type I	Myeloproliferative disorders	Sarcoidosis
		*CD53; LAMA3;*			
		*LAMA4; IL174A;*	Diabetic nephropathies	Crohn's disease	Carcinoma (multiple types)
		*HABP2; KLKB1*			
			Autosomal dominant polycystic kidney	Sjogren's syndrome	Gout
			Rhinitis	Nasal polyps	Telangiectasis
			Lesch-Nyhan syndrome	Arteriosclerosis	Genome instability

Metabolism	4	*P2RX2; LTF;*	Anoxia	Encephalitis	Rheumatoid arthritis
		*ACACB; PRKCZ*	Carcinoma (multiple types)	Diabetes mellitus type II	Glomberulonephritis

Nervous System	17	*CDK5RAP2;*	Microcephaly	Lipodystrophy	Rheumatoid arthritis
		*P2RX2;*	Anoxia	Mucolipidoses	Fragile X Syndrome
		*ST8S1A2; HTR1F;*	Schizophrenia	Pain	Liver cirrhosis
		*SCN10A; SLC28A2;*	Parkinson's disease	Asthma	Diabetes mellitus type I
		*MCOLN1; DLGAP4;*	Glioma	Acquired immunodeficiency	
		*SYT8; ATP2B3;*			
		*MAP1A; LAMA4;*			
		*MAP3K1; AHNAK;*			
		*AFF2; CASP7*			

Protein folding	4	*CHAF1A; GRPEL1;*	Aneurysm	Pulmonary fibrosis	Diabetes mellitus type I
		*HSPA4; EEF1A2;*			
			Carcinoma (multiple types)	Graft vs. host disease	Paralysis

Reproduction	13	*SPDY1; CENPI;*	Gastrointestinal diseases	Hyperaldosteronism	Genomic instability
		*BRCA2; NCOA4;*			
		*RBAK; CASC5;*	Carcinoma (multiple types)	Diabetes mellitus type II	Oligospermia
		*PRKCZ; MAP3K1*			
			anemia		

Signal transduction	19	*BRCA2; CASP7;*	Spontaneous abortion	Arteriosclerosis	Dilated cardiomyopathy
		*NCOA6;IL7R;*			
		*CYCS; BAMBI;*	Carcinoma (multiple types)	Hypertension	Asthma
		*KLKB1; IL17RA;*			
		*MAP3K1; DSG2;*	Huntington's disease	Severe combined immunodeficiency	Sarcoidosis
		*PRKCZ; IFI44;*			
		*IL18R1; HABP2;*	Thrombocytopenia	Multiple sclerosis	Diabetes mellitus type I
			Rheumatoid arthritis	Alzheimer's disease	Arrhythmogenic right ventricular dysplasia

Transcription	8	*NCOA6; BRCA2;*	Retinoblastoma	Oculopharyngeal muscular dystrophies	Rheumatoid arthritis
		*MAP3K1; TAF3;*			
		*PABPN1*	Spontaneous abortion	Aneuploidy	Deglutition disorders
			Carcinoma (multiple types)	Infertility	

Transport	11	*KCNH3; LTF;*	Anemia	Genomic instability	Liver cirrhosis
		*MSH2;NCOA4;*	Alzheimer's disease	Crohn's disease	Obesity
		*PPARGC1B;*	Epilepsy	Diabetes mellitus type II	Barrett esophagus
		*ZNF217;*	Carcinoma (multiple types)	Gastritis	progeria
		*ME2;P2RX2;ANK3;*			
		*LATS1; MAT2A*			

A list of genes containing probably or possibly damaging nsSNPs as predicted by PolyPhen-2 was submitted for network analysis in GeneGo. Characterization of the genes containing nsSNPs is achieved by listing the general cellular process (column 1), the total number of deleterious nsSNPs in the genes that GeneGo annotated (column 2), the genes associated with the cellular process (column 3), and the specific diseases that have been associated by GeneGo with the genes in question (columns 4-6).

Finally, we compared the list of validated Indian-origin rhesus macaque SNPs to the locations of known SNPs in the human genome (dbSNP, build 132). The evolutionary lineage leading to rhesus macaques diverged from the human lineage about 22-26 million years ago, and the two genomes have diverged more than 6% in overall DNA sequence since that time [23]. Consequently, except in unusual loci such as HLA, homologous basepair positions that are polymorphic SNPs in both species are highly unlikely to represent shared ancestral polymorphism that has been retained for more than 20 million years in both lineages. Rather, most shared SNP positions will reflect parallel mutational events that created variation at the same site in both species. We converted all 3,038,166 validated rhesus SNPs from this study to hg19 human coordinates, and 2,775,850 of those SNPs successfully converted using the Galaxy hosted UCSC liftOver tool. Of these converted SNPs, 90,086 exactly matched the location of human SNPs in dbSNP Build 132, representing a 3.2% overlap. Given that there are 29,100,846 human SNPs (chromosomes 1-22, X) in build 132, about 1% of the human genome, if the specific locations of SNPs in the human and rhesus genomes were entirely uncorrelated then we would only expect about one-third as many polymorphic basepair positions to be shared in the two species. This correlation between the locations of SNPs in rhesus monkeys and humans is almost certainly influenced in part by constraints on which sites within each genome can tolerate polymorphism. But other mechanisms, such as correlations in mutation rates at homologous sites across primate genomes, may also be involved.

## Discussion

**Figure 4:**
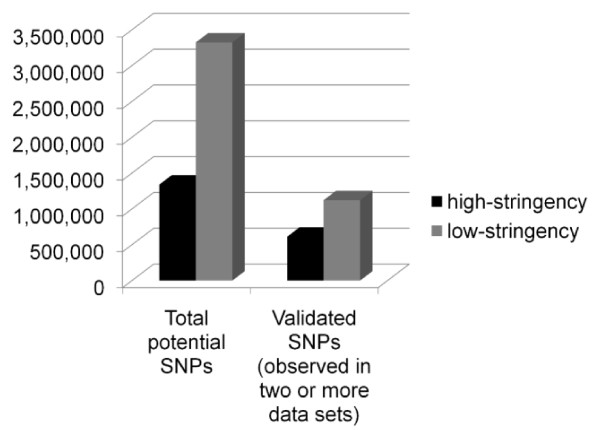
**Low stringency vs. high stringency SNP call validation effectiveness**. Validation efficiencies cannot be determined using existing validated SNP data sets, as only 777 SNPs are currently available for rhesus macaque in dbSNP, most of which are polymorphic between subspecies (Chinese to Indian) rather than within subspecies (ie: Indian to Indian). We observed improved validation efficiency using low-stringency SNP calls rather than high-stringency. Both high and low stringency SNP calls were obtained for the reference animal (17573) mate-pair sequence data. The percentage of total SNPs validated in the low-stringency SNP set was slightly less (33.8%) than that observed in the high stringency SNP set (45.7%). In absolute numbers, however, there were 1.8X more SNPs validated from the low stringency SNP calls compared to the high stringency SNP calls.

In this study, we validated 3,038,166 SNPs and identified an additional 11,382,666 potential SNPs for the Indian-origin rhesus macaque. Both Chinese and Indian-origin rhesus macaques are used in biomedical research, but Indian-origin animals are more prevalent. Furthermore, the rhesus macaque reference genome was produced using an individual of Indian-origin [23]. At the time we began this study, very few SNPs or other variants were confirmed or validated in the rhesus genome. By taking advantage of the falling cost of next-gen resequencing and utilizing all of the existing sequence data available, we were able to validate a large genome-wide series of SNPs. Different sequencing technologies exhibit distinct probabilities of different sequencing errors that will increase false positive rates in calling novel SNPs [38]. SNPs validated in a single individual using multiple sequencing technologies avoid this problem, thus increasing confidence in the SNP calls common to the two data sets. In addition to inherent sequencing chemistry concerns, low coverage sequencing can suffer from read coverage limitations, creating false negative data because the SNP calling software will discard significant proportions of legitimate SNPs due to low coverage (Figure 4). For this reason, we chose to utilize reduced stringency SNP calls from corona_lite and AtlasSNP2. Our methodology minimizes both false positive and false negative SNP calls by using reduced-stringency SNP calling standards but requiring that each SNP location and both alleles be confirmed in data obtained from different animals and/or different chemistries. In addition, most of our comparisons utilized comparisons of SNP data sets that were called by different algorithms. Studies of human population variation from the 1000 Genomes Project [39] have shown that utilizing the intersection of different SNP calling methods to hone in on real SNP data reduces the false positive SNP error rate by 30-50%. This validated SNP list represents a substantial increase in the number of known rhesus SNPs, and therefore is an important resource for research involving this species. These data will facilitate the discovery of functional SNPs, the development of new disease models, and genetic linkage studies, as well as providing a valuable resource for colony management.

The most interesting SNPs in any data set are those that directly alter biological function, but these are also the most challenging to identify. While functional SNPs can be in coding (non-synonymous or splice site SNPs) or non-coding regions (non-coding RNAs, regulatory regions), the simplest SNPs to evaluate for function are in coding regions. We took advantage of the close evolutionary relationship between rhesus macaques and humans to generate hypotheses about the functionality of the non-synonymous SNPs we identified. With 411 SNPs that are predicted to be probably damaging by PolyPhen-2 [36], we have begun extending the information concerning potentially significant functional variation in this species. Out of these SNPs, several occur in genes associated with behavior (*Scn10A*), metabolism (*Ppgca1b*), cancer (*Brca2*), and immune response (*Map3k1*, *OAS2*, *IL18R1*).

Our study has identified substantial genetic variation among Indian-origin rhesus monkeys. Previous information [24] shows that separate SNP identification efforts are required in Chinese-origin and Indian-origin monkeys. Only about one-third of SNPs that are polymorphic in one population are also polymorphic in the other [27]. Thus, the information we report here is potentially helpful to investigators using Chinese-origin rhesus macaques, but additional work to discover SNPs in that population is warranted, just as information concerning variability among Chinese-origin animals is only partially relevant to our study population.

While this study produced greater than 3,000 fold more SNPs than had previously been known in rhesus macaques (both Indian and Chinese), it is not exhaustive. Roughly 25% of each potential SNP list was validated using our methodology (Figure S1, Additional file 1). Further validation of the remaining potential SNPs may proceed either through additional sequencing of the large data sets, or by sequencing additional animals. Currently, there are more than 37,821,067 human SNPs in dbSNP. Not only does that predict a lower bound of ~34 million rhesus macaque SNPs yet to be identified, but the hypothesized increased genetic variation in rhesus macaques indicates that we may identify many more SNPs than have been validated in humans. This study focused upon the genome-wide identification of SNPs in an effort to meet a wide variety of needs in the primate research community. Future work highlighting coding variants could take advantage of deep re-sequencing of exomes or re-sequencing specific loci where prior information suggests a potential role in disease or phenotype.

## Conclusions

Our study identified more than 14 million potential single nucleotide variants and validated ~3 million of these variants. Various strategies can be used to detect and validate SNPs in any given species. We present an approach that combines different types of next-gen sequence data with low stringency bioinformatic filters, and relies upon differences among individuals and across sequencing chemistries to validate variants. Validated SNP coverage was relatively even throughout the genome. Additionally, a proportion of validated SNPs are predicted to be probably damaging in rhesus homologues of genes that are known to contribute to disease in humans. This increase in information concerning genetic variation in Indian-origin rhesus macaques opens up many new opportunities for the research community in evolutionary studies, the use of rhesus macaques as models of human disease, and studies of primate diversity.

## Materials and methods

### Ethics Statement

All relevant animal procedures in this project were approved by the appropriate Institutional Animal Care and Use committees from the Southwest National Primate Research Center or the University of Wisconsin, and comply with the federal regulations. Genomic DNA for sequencing was obtained either from frozen tissues collected previously as part of other research projects or from blood samples collected from live animals by venupuncture, using appropriate anesthetics.

### Data sources

We began our analysis with the Sanger sequence data generated as part of the original rhesus macaque genome project [23]. A single animal (animal ID 17573) from the Southwest National Primate Research Center (San Antonio, TX) Indian-origin rhesus colony was sequenced to approximately 5.2X coverage (18.4 Gb of raw sequence) using Sanger whole genome shotgun methods. Additional details of that reference genome analysis have been published previously [23]. From this Sanger data, approximately 4.3 million basepair positions were called as potentially heterozygous using SNPdetector [40] (Houston, TX). We obtained additional genomic DNA from animal 17573 and created two libraries for SOLiD sequencing, one library for fragment sequencing and one for mate-pair sequencing (Table S1, Additional file 3). Using published methods [41,42] (50bp fragment (SRA accession: SRX029055) and 25 × 25bp mate pair data (SRA accession: SRX029056 )) we generated 5X fragment sequence coverage and approximately 8X mate-pair sequence coverage. The proportion of matching beads to total beads was: 49.52% for the fragment library and 55.22% for the mate pair library (F3+R3). Following the re-sequencing of the original reference animal, we selected two unrelated Indian-origin rhesus macaques (animal ID's r1766 (SRA accession: SRX029057) and r02120 (SRA accession: SRX029058)) from the population at the Wisconsin National Primate Research Center (University of Wisconsin, Madison). Each DNA sample was used to produce fragment sequencing libraries, and each library sequenced to low coverage (50bp HiDRA, r1766 to 5.8X, r02120 to 3.3X). The ratio of matching beads to total beads was 58.64% and 49.89%, respectively. The library for r02120 exhibited a decreased degree of unique beads relative to r1766 and 17573, but did produce a substantial amount of high-quality data.

We also downloaded additional 454 sequence reads from NCBI (26.2 Mb) produced during the original rhesus sequencing project [23] from a comparison of rhesus macaque sub-species (8 Chinese-origin and 8 Indian-origin unrelated rhesus macaques). SNPs were called using these reads and the default settings for AtlasSNP2 [43] without filtering for coverage. Sub-species comparison read data was provided at 0.5X coverage [23]. 142,781 potential SNPs were identified in the sub-species comparison data set.

Several lists of SNP calls were generated previously (Table 3). MamuSNP provided a list of 22,892 potential SNPs [25,26]**(**http://mamusnp.ucdavis.edu/query.php[44]). ENCODE consisted of a list of 1,672 validated SNPs out of which 1,467 have been previously described [24]. Additional validated SNPs were downloaded from dbSNP (http://www.ncbi.nlm.nih.gov/projects/SNP/[16]) and MonkeySNP (http://monkeysnp.ohsu.edu/snp/[45]), for a total of 765 SNPs at the time of download. Overlap was almost exact (763 of 765) between these two data sources and the data set will hereafter be referred to as dbSNP.

**Table 3:** Data sources

Chemistry	Name	DNA source	SNP calling software	References
Sanger	Assembly	Animal 17573 ♀	SNPdetector	Gibbs, et al. (2007) [23], Wheeler (pers. comm.)

Sanger	ENCODE	47 pooled individuals	SNPdetector	Hernandez, et al. (2007) [24]

SOLiD	17573 fragment	Animal 17573 ♀	Corona_lite	

SOLiD	17573 mate-pair	Animal 17573 ♀	Corona_lite	

SOLiD	r1766	Animal r1766 ♀	Corona_lite	

SOLiD	r02120	Animal r02120 ♂	Corona_lite	

454	Sub-species comparison	16 pooled individuals	AtlasSNP	Gibbs, et al. (2007) [23]

454	MamuSNP	~7 pooled individuals	Unknown	Malhi, et al. (2007) [25], http://mamusnp.ucdavis.edu/query.php[44]

unknown	dbSNP/MonkeySNP	Unknown, >30 estimated	Unknown	http://monkeysnp.ohsu.edu/snp/[45], http://www.ncbi.nlm.nih.gov/projects/SNP/[16]

Each data source is displayed with the appropriate chemistry used, DNA source (if known) and SNP calling software.

### SNP calling and validation methods

Identification of specific bases that are heterozygous in a given individual was accomplished using different methods for different sequencing technologies. The original Sanger read data for the reference animal was searched for SNPs (i.e. heterozygous basepair positions) using SNPdetector [40]. For the SOLiD read data, corona_lite v4.0 r2.0 (Life Technologies, Carlsbad, CA) was used. SNPs were called in the sub-species comparison data using AtlasSNP2 [43] as described above.

We considered all basepair positions called as heterozygous in any one dataset as "potential SNPs." In order to classify a given SNP as "validated," we required the specific base pair position be called as an identical heterozygote in two independent datasets. This could mean either observing that position as heterozygous in the same individual using two different sequencing methods, or observing that position to be heterozygous, with identical alleles, in two different animals. Two complimentary methods were used to identify SNPs in the data sets described above: positional-allele comparison and e-genotyping.

Positional-allele comparison relies upon parameters of sequence quality and read coverage as implemented in various SNP calling programs (in this study we used SNPdetector, corona_lite, and AtlasSNP2). This method compares lists of identified potential SNPs by chromosome, base pair location, and both alleles. The comparison method can identify multiple SNPs clustered close together or those near to breakpoints in the reference sequence. But this method has a potentially higher false negative rate compared with e-genotyping, due to the relative stringency of the SNP calling programs used upstream. To reduce false negative calls, we used stringency thresholds somewhat lower than those used in some other studies. Our settings generally retained potential SNPs with read quality scores equivalent to a Phred score of 20 and a minimum of 2 reads covering each variant allele call.

E-genotyping is a novel approach that takes advantage of an *a priori *defined set of potential SNPs, and then tests raw reads (color-space or sequence space data) directly for exact probe matches to the previously defined SNP location and sequence. A probe region consists of 15 bases on each side of the potential SNP. These flanking regions must be pre-defined, and must match the target reads in the new sequence dataset exactly, with different reads providing exact matches to the two expected alleles in the potential SNP position. This novel method of validating potential SNPs in a new read set is restricted by the exact matching parameter that confers the high specificity. When e-genotype was used to screen human 1000 Genomes read data for known SNPs (from dbSNP) in the same individual, the error call rates were extremely low for miscalled homozygotes (called as wrong homozygote (0.002%)) and heterozygotes (called as wrong heterozygote (1.01%) or homozygote (1.52%)) (Figure S3, Additional file 4). dbSNP concordance in human data from the 1000 Genomes pilot study [39] using e-genotype indicates that the total miscall rate is ~2.6% (Figure S3, Additional file 4). When extremely high coverage or low coverage SNPs (delineated by dotted lines on figure) are removed from consideration (high coverage SNPs are typically non-specific and low coverage SNPs are often miscalled hets where coverage was simply insufficient to detect both alleles), the miscall rate is calculated to be 1.6%. Because of the stringency of probe placement, e-genotype has much reduced effective read coverage for any SNP, but of the SNPs that are called by e-genotype, the probability of false positive calls is extremely low. E-genotype has significantly reduced power to detect clustered SNPs, especially in linkage disequilibrium, when they fall within the 31 base pair probe region. E-genotype is unable to identify SNPs located within 15 base pairs of gap regions in the reference sequence or chromosome ends.

We developed a perl pipeline to compare chromosome, base pair locations, and both alleles for all pair-wise comparisons of SNP lists. At least one read of sufficient quality (equivalent to a minimum Phred score of 20) for each allele in each data set was required for a positive result, and all instances with more than 200 reads covering an allele were removed irrespective of average read coverage for the data set, due to presumed mis-mapping or non-unique mapping. Once these comparisons were completed, all of the validated SNPs were appended in a single file and duplicates were removed by chromosome/base-pair determination.

E-genotyping (http://is04607.com/~drio/egenotype/[29]) was performed on all the SOLiD read sets from animals 17573, r1766 and r02120. The probe sets were created from: a) the Sanger data from the original reference animal (17573), b) the novel SNP calls from corona_lite for r1766 and r02120, or c) the reads produced using Roche 454 methods on unrelated animals in the original rhesus genome paper [23]. All the probe sets were checked for unique mapping locations by mapping to the reference genome assembly prior to running e-genotype on experimental read sets. E-genotyping results with at least one read for each allele were considered to be positive when comparing data from different chemistries.

We annotated the SNPs using a Java tool that accessed Ensembl (build 57, March 2010). Annotation of SNPs included the genomic placement (intronic, intergenic, synonymous coding, nonsynonymous coding, 5'UTR, 3'UTR, or splice site) for all applicable transcripts. If a SNP was determined to fall in a genic region (coding and non-coding), the following annotations were added as applicable: gene name, gene function, codon, reference amino acid, variant amino acid, protein position.

All of the validated SNPs have been submitted to dbSNP (http://www.ncbi.nlm.nih.gov/projects/SNP/[16]) (ss numbers available in Table S2, Additional files 5, 6, 7, 8, 9 and 10). Unvalidated potential SNPs will be available at our lab Genboree site (http://genboree.org/java-bin/project.jsp?projectName=Rhesus%20SNPs%20using%20Next-Gen%20Sequencing&isPublic=Yes) [30].

### Polyphen-2 analysis and GeneGo analysis

Non-synonymous SNPs (nsSNPs) from this validated list of rhesus macaque SNPs were converted to human genome (hg18) co-ordinates using the UCSC liftOver tool from Genboree Galaxy and then analyzed for possible functional significance using PolyPhen-2 (http://genetics.bwh.harvard.edu/pph2/[35]) [36]. All SNPs that did not convert cleanly to unique locations in hg18, or where the human reference allele at that location matched the rhesus variant allele, were removed from further analysis. The resulting list of human homologues of our rhesus nsSNPs was submitted as a batch file for HumDiv model analysis in PolyPhen-2. Polyphen-2 utilizes both homologous sequence alignment as well as known protein 3D crystal structures to predict the potential effect of any given polymorphism upon protein function. Polymorphisms are tested for the potential damaging effect upon protein function by normalization to known human deleterious alleles in UniProt and use of a naïve Bayes classifier. All genes containing SNPs that were classified as damaging (probably or possibly) in the PolyPhen-2 output were submitted to GeneGo (St. Joseph, MI, http://www.genego.com[37]) for network analysis. Each gene was associated with a generalized function and proposed disease network.

## Competing interests

The authors declare that they have no competing interests.

## Authors' contributions

JR, GLF and RG contributed to project conception. GLF, FY, JGR, DMM, AM, DAW, KCW, RG and JR contributed to experimental design and approach. Software tools were created by: GLF (perl comparison tool pipeline), DRD and JGR (e-genotype), FY (AtlasSNP), RAH and AM (Genboree), YR (SNPdetector). Experiments were performed by GLF, MR, DRD, DC, and RAH. Data was analyzed by: GLF, MR, DRD, DC, YR. Resources to generate data were provided by: DMM and RG (library and sequencing resources), YR and DAW (initial list of reference animal heterozygotes), SS and NHK (rhesus DNA), JR (rhesus DNA). The manuscript was prepared by GLF and JR. All authors read and approved the final manuscript.

## Supplementary Material

Additional file 1 **Figure S1, Additional file 1. Validation by data source**. Validation efficiencies cannot be determined using existing validated SNP data sets, as only 777 SNPs are currently available in dbSNP for rhesus macaque, most of which are polymorphic between subspecies (Chinese to Indian) rather than within a subspecies (Indian to Indian). We calculated the proportions of unique SNPs validated within each pairwise comparison of all 15 data sets. On average, we validated ~35% of the potential SNPs from each of the resequencing data sets. r02120 displays much higher rates of validation (~65%) compared to all other data sets, likely due to technical issues resulting in much lower coverage. The Sub-species comparison data set exhibited similar validation rates (~40%) to the resequenced data sets, which may be explained by the fact that this data set contained many more SNPs than the other non-resequenced data sets. The smaller data sets (MamuSNP, ENCODE, dbSNP, and all e-genotyping data sets) all displayed significantly lower validation rates (~10%) due both to these data sets being generated using both Chinese and Indian origin animals rather than only Indian animals and to the small number of SNPs in each of these sets. Click here for file

Additional file 2 **Figure S2, Additional file 2. Sanger read coverage for validated SNPs**. All SNPs that were found in common between r1766, r02120, and 17573 (306,782, **Figure 1B**) were tested for Sanger read coverage using the 17573 Sanger data by looking for the same location and allele SNP call in the Sanger output (red bars). Average genome-wide Sanger read coverage was 5.2X. We analyzed an identical number of randomly selected SNPs that were detected in only two of the three resequenced animals (blue bars) to test if there were differences in the distribution of read coverage for the two SNP data sets. The distributions are indistinguishable, however, and the proportions of tested SNPs exhibiting 11+ read coverage are not statistically different (3.8% for SNPs in all three animals, 3.6% for SNPs detected in only two animals). Click here for file

Additional file 3 **Table S1, Additional file 3. SOLiD sequencing statistics**. When using our new SOLiD data or prior data from the literature, our validation procedure only validated two alleles for any location, regardless of the data sets being compared. However, for some SNPs, neither of the two validated alleles matched the published reference allele. Some (8) of these are likely due to Indian-origin vs. Chinese-origin differences. For the vast majority, however, there are two possibilities, each of which explains an undefinable proportion of these SNPs: (1) a proportion of the reference allele (Sanger) calls are wrong, and (2) a small proportion of the apparent three allele SNPs represent true SNPs with three valid alleles in Indian-origin rhesus macaques. Click here for file

Additional file 4 **Figure S3, Additional file 4. dbSNP concordance of e-genotype**. One human sample of SOLiD data (26X coverage) from the 1000 Genomes pilot data was analyzed for the ~1 million SNPs identified in the project for that sample. Color coding for the bars as well as the lines is as follows: good homozygous calls (blue), heterozygotes called as homozygotes (yellow), homozygotes called as the wrong homozygote (grey), good heterozygote calls (green), and erroneously called heterozygotes (red). The total miscall rate was ~2.6%. Excluding very high and very low coverage errors (shown outside of dotted lines, due to bad heterozygous SNP calls from repetitive regions or coverage too low to detect heterozygotes, respectively) the miscall rate was determined to be 1.6%. Overall probe coverage was reduced to 8.2X due to the extremely stringent requirement of exact matching of probes for the full 31 bp probe length. Percentages of homozygous or heterozygous calls that fit into each category of good or error calls were linearly graphed relative to probe coverage, indicating that errors were much more likely at very high and very low probe coverage, while good calls were most likely in intermediate coverage ranges. Click here for file

Additional file 5 **Table S2, Additional file 5. Part 1 of dbSNP ss numbers for validated rhesus macaque SNPs**. The first part of six of a table listing of all of the ss numbers from submission of the validated rhesus macaque SNPs to dbSNP. The ss numbers are not contiguous throughout the full set of 3,038,166 SNPs. Click here for file

Additional file 6 **Table S2, Additional file 6. Part 2 of dbSNP ss numbers for validated rhesus macaque SNPs**. The second part of six of a table listing of all of the ss numbers from submission of the validated rhesus macaque SNPs to dbSNP. The ss numbers are not contiguous throughout the full set of 3,038,166 SNPs. Click here for file

Additional file 7 **Table S2, Additional file 7. Part 3 of dbSNP ss numbers for validated rhesus macaque SNPs**. The third part of six of a table listing of all of the ss numbers from submission of the validated rhesus macaque SNPs to dbSNP. The ss numbers are not contiguous throughout the full set of 3,038,166 SNPs. Click here for file

Additional file 8 **Table S2, Additional file 8. Part 4 of dbSNP ss numbers for validated rhesus macaque SNPs**. The fourth part of six of a table listing of all of the ss numbers from submission of the validated rhesus macaque SNPs to dbSNP. The ss numbers are not contiguous throughout the full set of 3,038,166 SNPs. Click here for file

Additional file 9 **Table S2, Additional file 9. Part 5 of dbSNP ss numbers for validated rhesus macaque SNPs**. The fifth part of six of a table listing of all of the ss numbers from submission of the validated rhesus macaque SNPs to dbSNP. The ss numbers are not contiguous throughout the full set of 3,038,166 SNPs. Click here for file

Additional file 10 **Table S2, Additional file 10. Part 6 of dbSNP ss numbers for validated rhesus macaque SNPs**. The sixth part of six of a table listing of all of the ss numbers from submission of the validated rhesus macaque SNPs to dbSNP. The ss numbers are not contiguous throughout the full set of 3,038,166 SNPs. Click here for file
