# Supplementary material for: Characterization of single-nucleotide variation in Indian-origin rhesus macaques (Macaca mulatta)
Source: BMC Genomics. 2011 Jun 13;12:311. doi: 10.1186/1471-2164-12-311 (PMC3141668; doi:10.1186/1471-2164-12-311)
Supplement: Additional file 1 — Figure S1, Additional file 1. Validation by data source. Validation efficiencies cannot be determined using existing validated SNP data sets, as only 777 SNPs are currently available in dbSNP for rhesus macaque, most of which are polymorphic between subspecies (Chinese to Indian) rather than within a subspecies (Indian to Indian). We calculated the proportions of unique SNPs validated within each pairwise comparison of all 15 data sets. On average, we validated ~35% of the potential SNPs from each of the resequencing data sets. r02120 displays much higher rates of validation (~65%) compared to all other data sets, likely due to technical issues resulting in much lower coverage. The Sub-species comparison data set exhibited similar validation rates (~40%) to the resequenced data sets, which may be explained by the fact that this data set contained many more SNPs than the other non-resequenced data sets. The smaller data sets (MamuSNP, ENCODE, dbSNP, and all e-genotyping data sets) all displayed significantly lower validation rates (~10%) due both to these data sets being generated using both Chinese and Indian origin animals rather than only Indian animals and to the small number of SNPs in each of these sets. [file 1471-2164-12-311-S1.PDF]

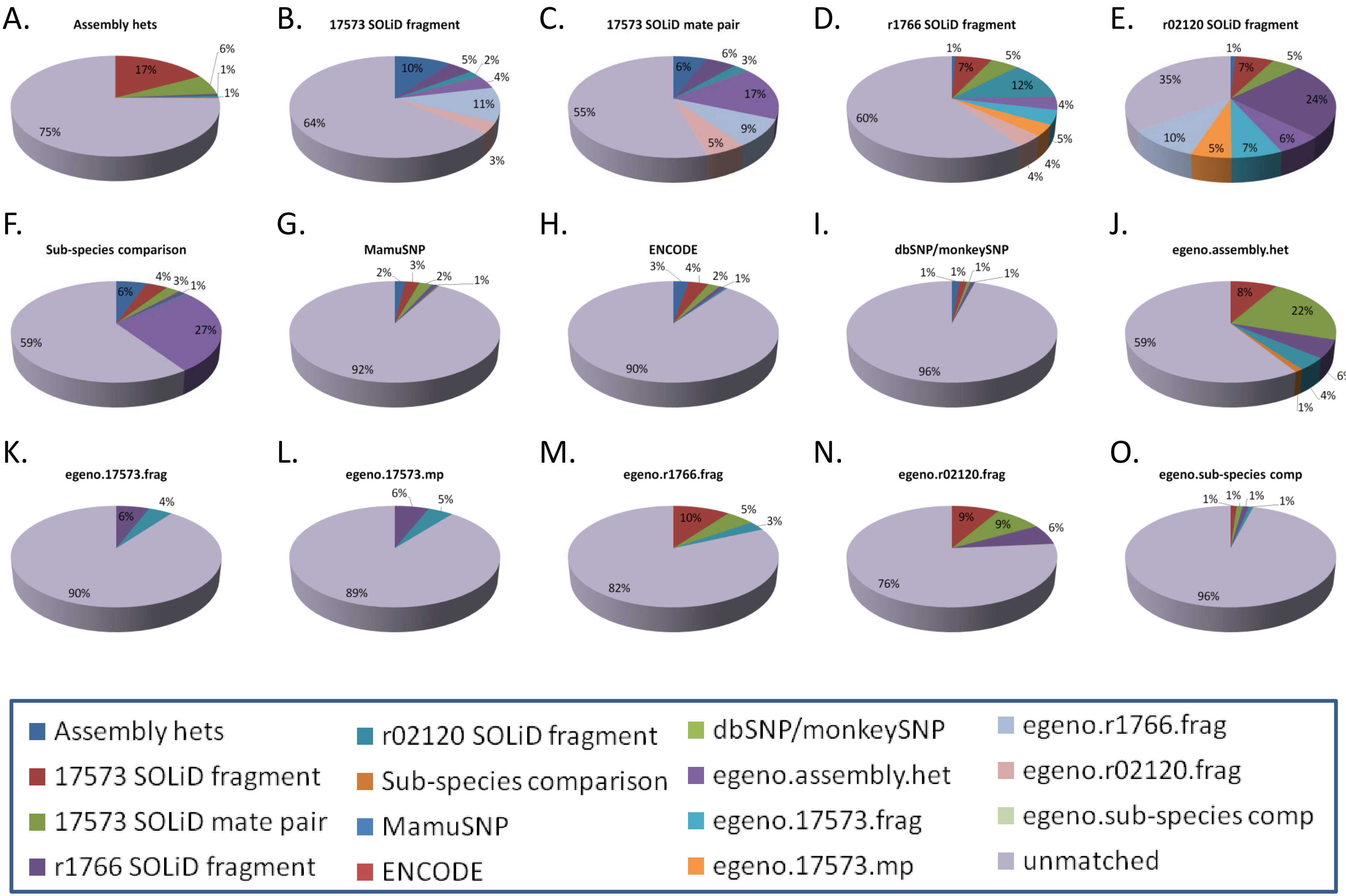
A.
Assembly hets
17%
6%
1%
1%
75%
B.
17573 SOLiD fragment
10%
5%
2%
4%
11%
64%
3%
C.
17573 SOLiD mate pair
6%
6%
3%
17%
55%
9%
5%
D.
r1766 SOLiD fragment
1%
7%
5%
12%
4%
60%
5%
4%
4%
E.
r02120 SOLiD fragment
1%
7%
5%
35%
24%
10%
6%
5%
7%
F.
Sub-species comparison
6%
4%
3%
1%
27%
59%
G.
MamuSNP
2%
3%
2%
1%
92%
H.
ENCODE
3%
4%
2%
1%
90%
I.
dbSNP/monkeySNP
1%
1%
1%
1%
96%
J.
egeno.assembly.het
8%
22%
59%
6%
4%
1%
K.
egeno.17573.frag
6%
4%
90%
L.
egeno.17573.mp
6%
5%
89%
M.
egeno.r1766.frag
10%
5%
3%
82%
N.
egeno.r02120.frag
9%
9%
6%
76%
O.
egeno.sub-species comp
1%
1%
1%
1%
96%
Assembly hets
17573 SOLiD fragment
17573 SOLiD mate pair
r1766 SOLiD fragment
r02120 SOLiD fragment
Sub-species comparison
MamuSNP
ENCODE
dbSNP/monkeySNP
egeno.assembly.het
egeno.17573.frag
egeno.17573.mp
egeno.r1766.frag
egeno.r02120.frag
egeno.sub-species comp
unmatched

Assembly hets

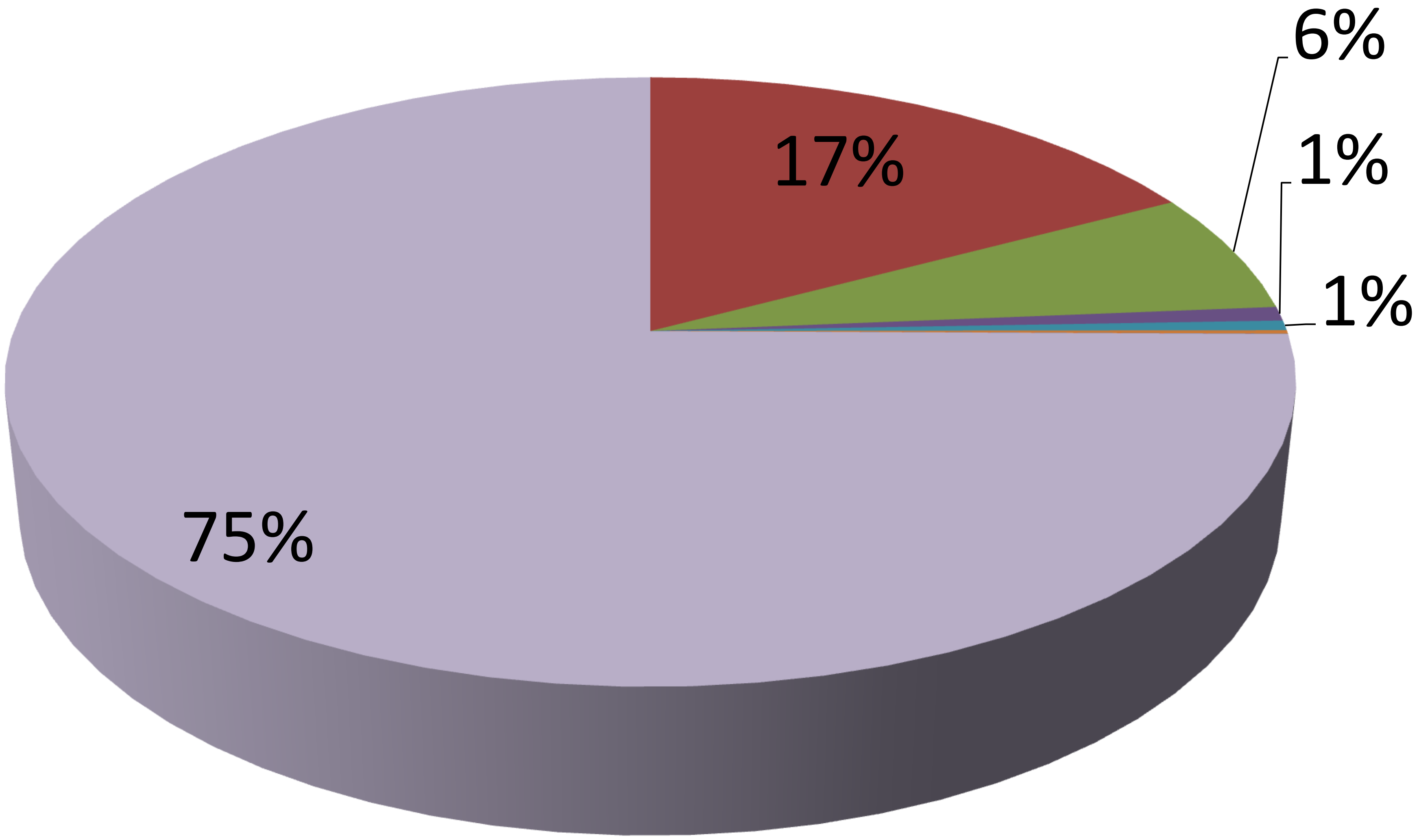

17573 SOLiD fragment

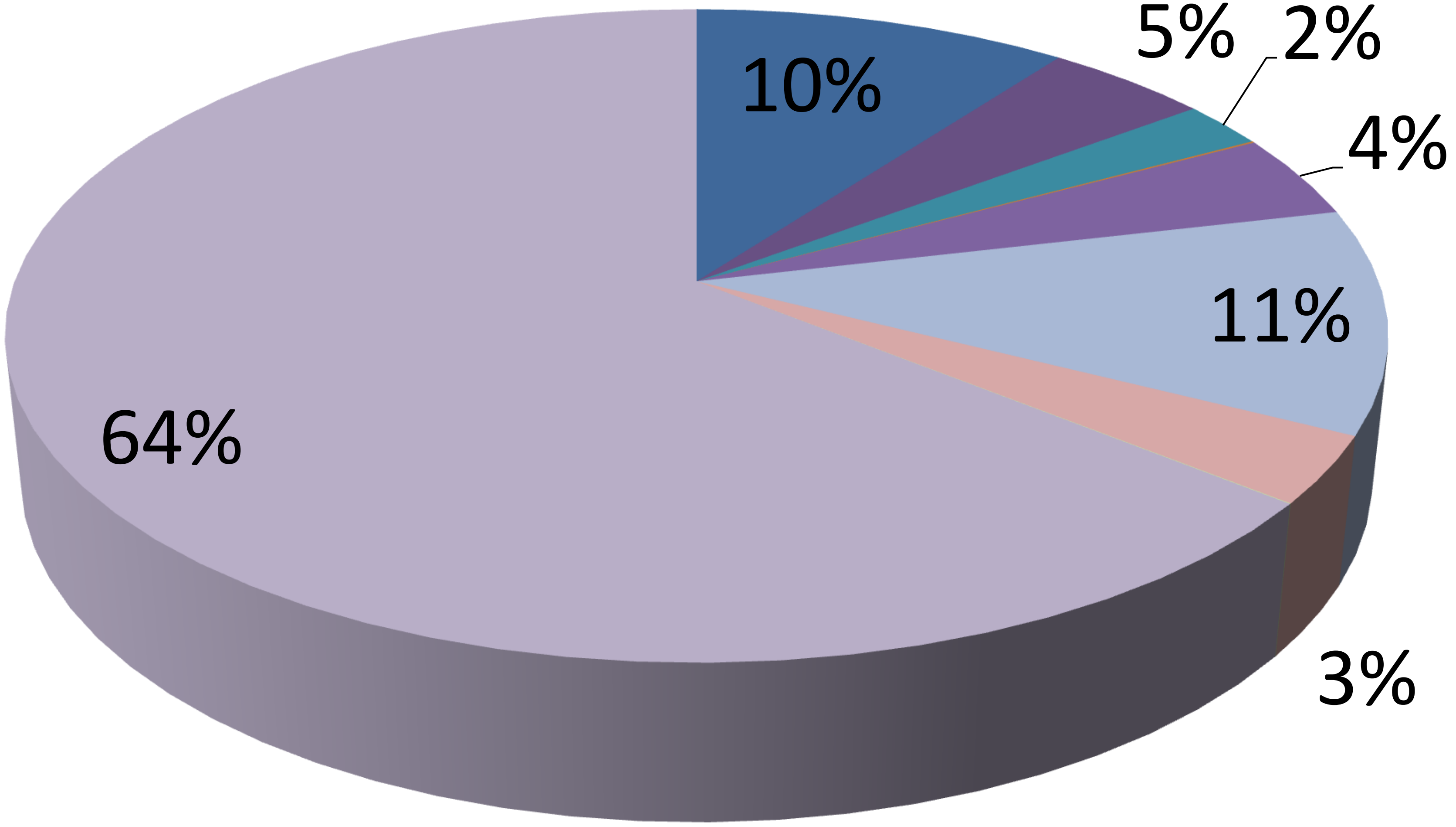

17573 SOLiD mate pair

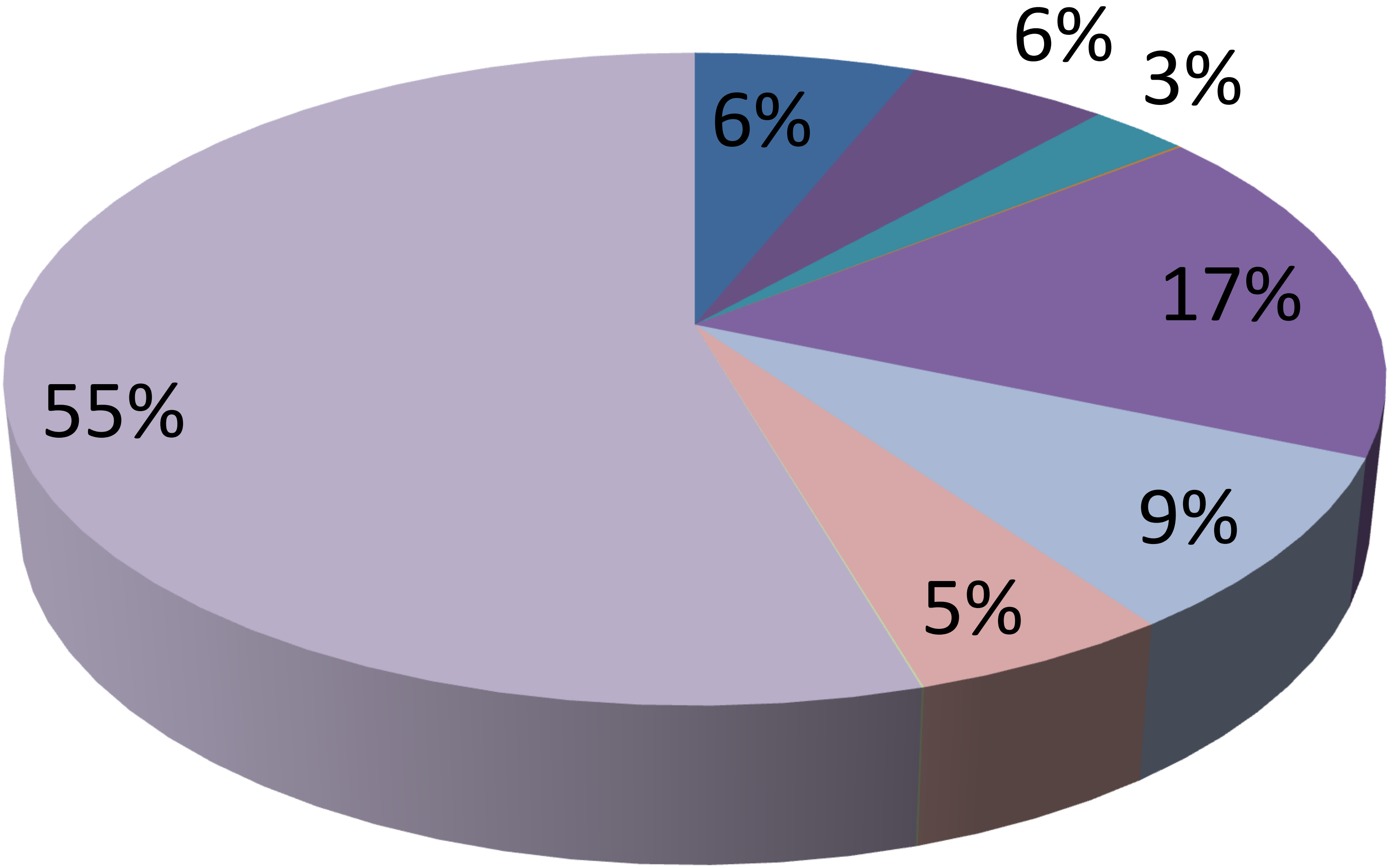

r1766 SOLiD fragment

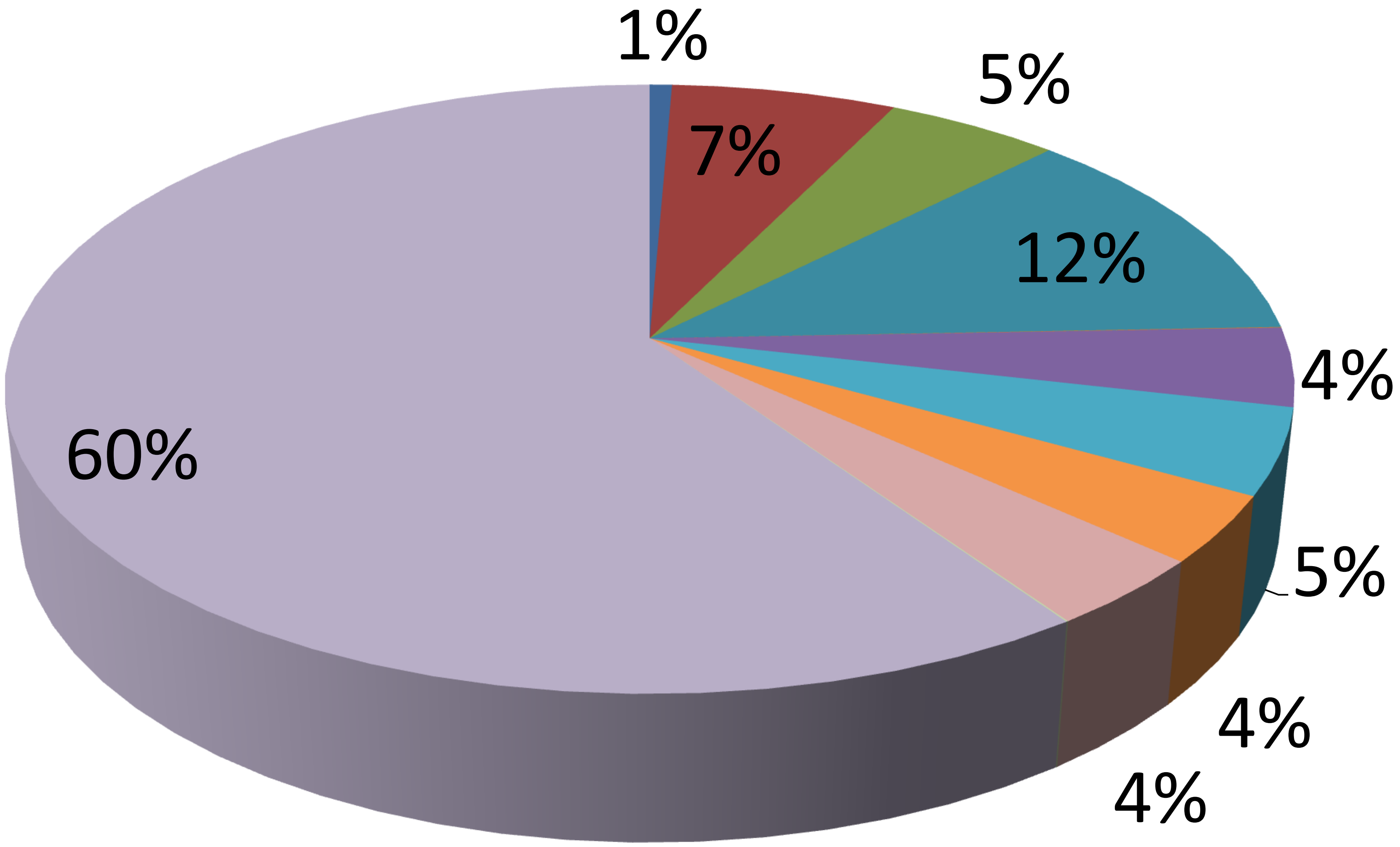

r02120 SOLiD fragment

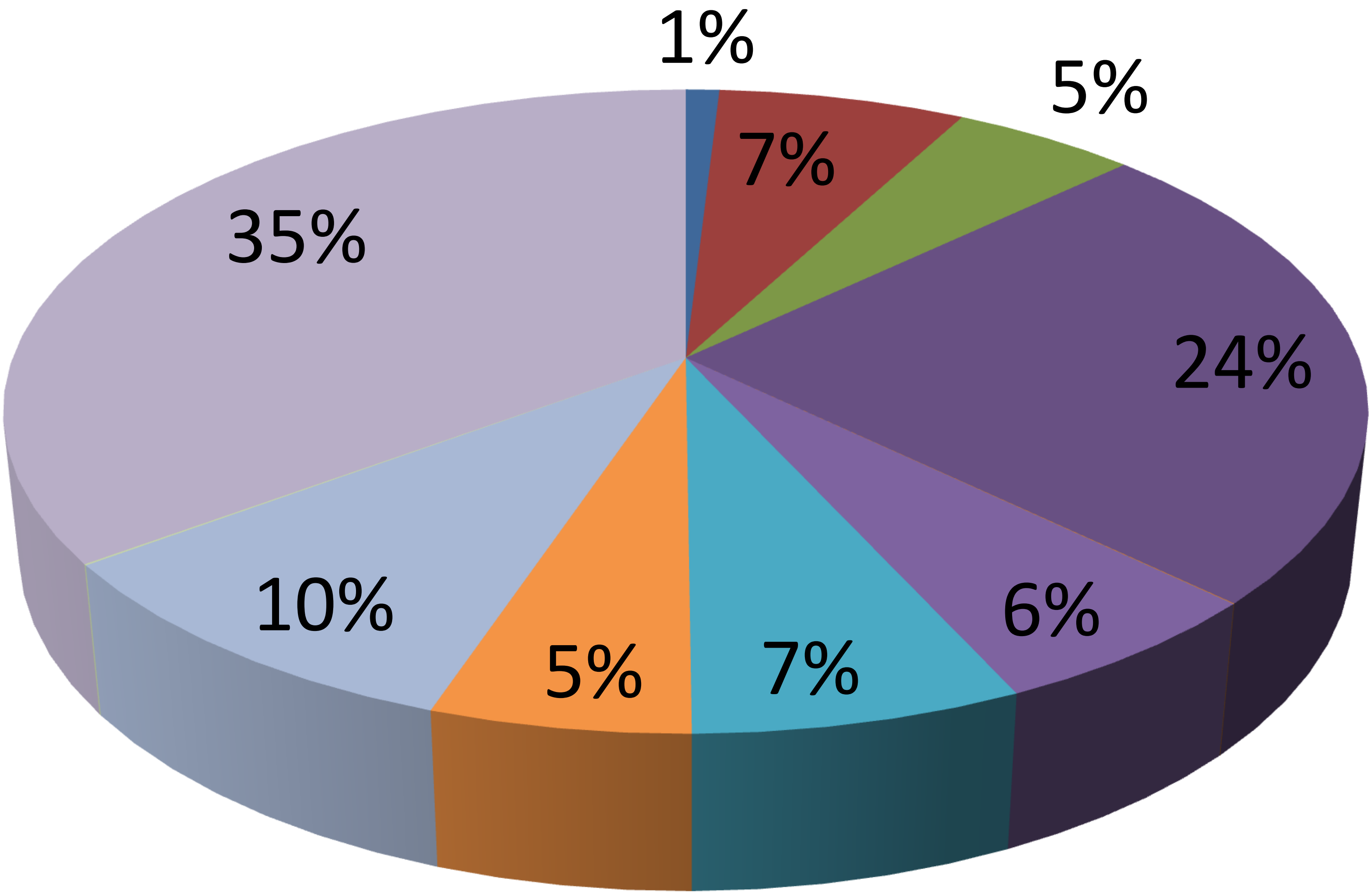

Sub-species comparison

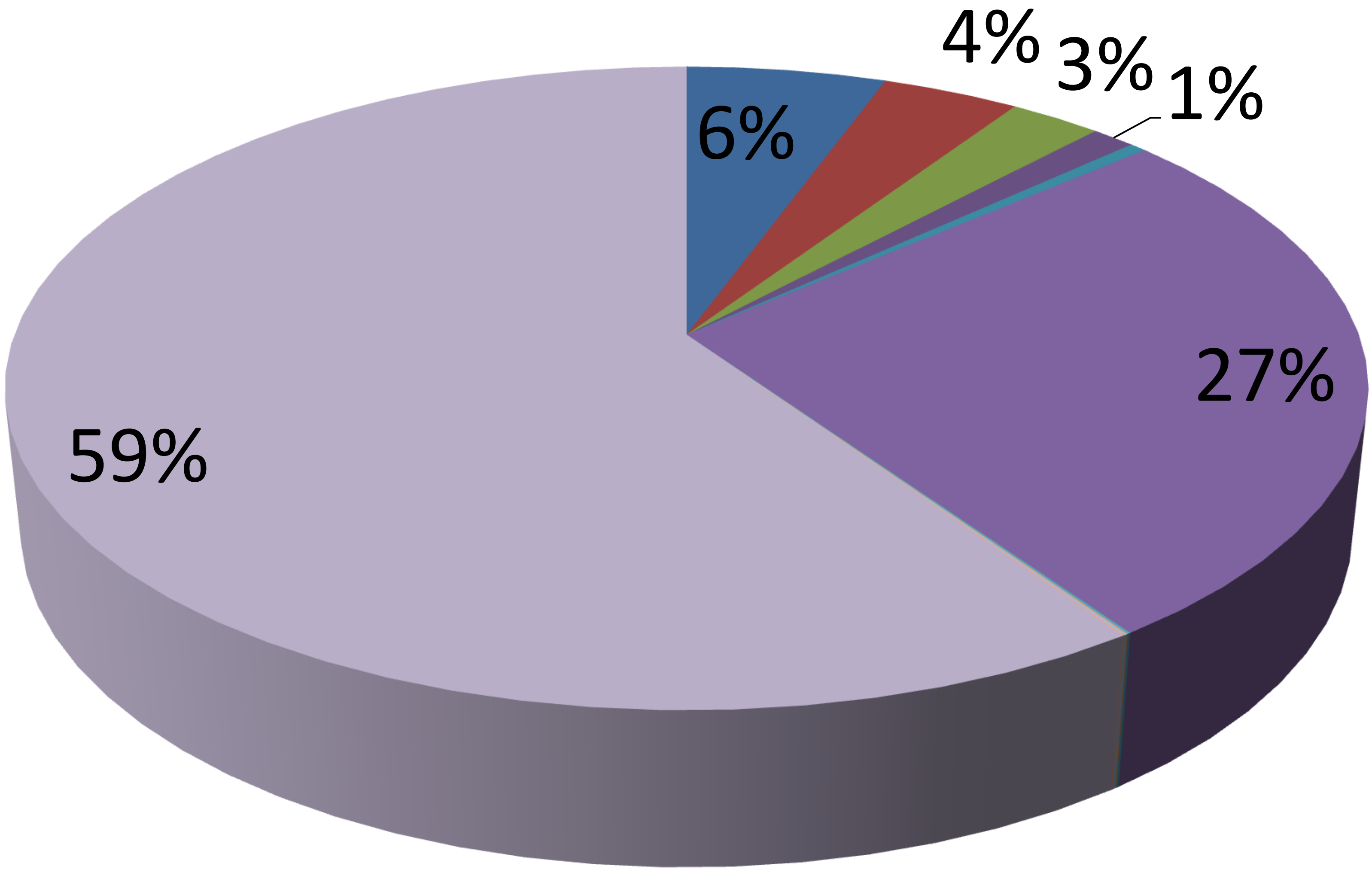

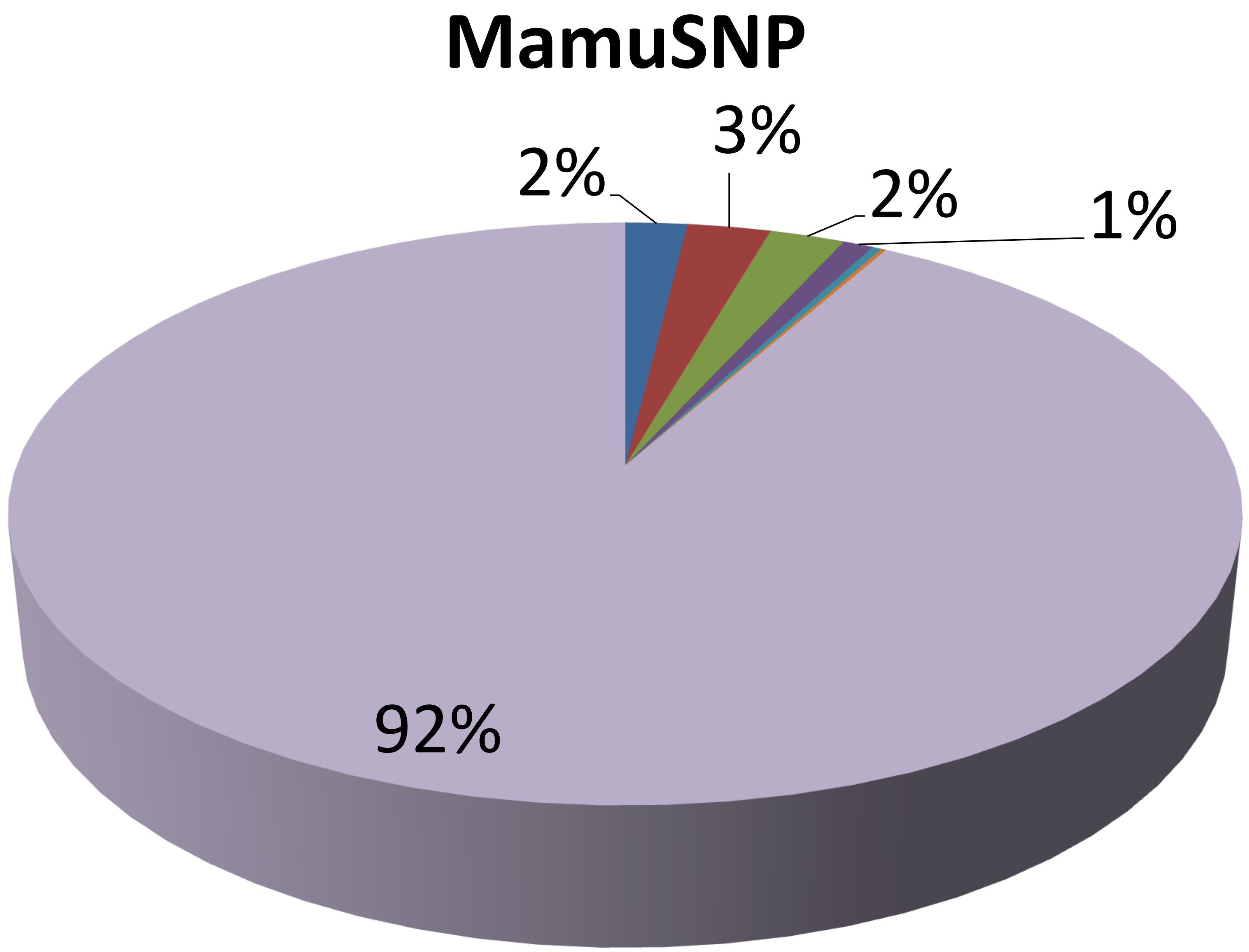
MamuSNP
2%
3%
2%
1%
92%

ENCODE

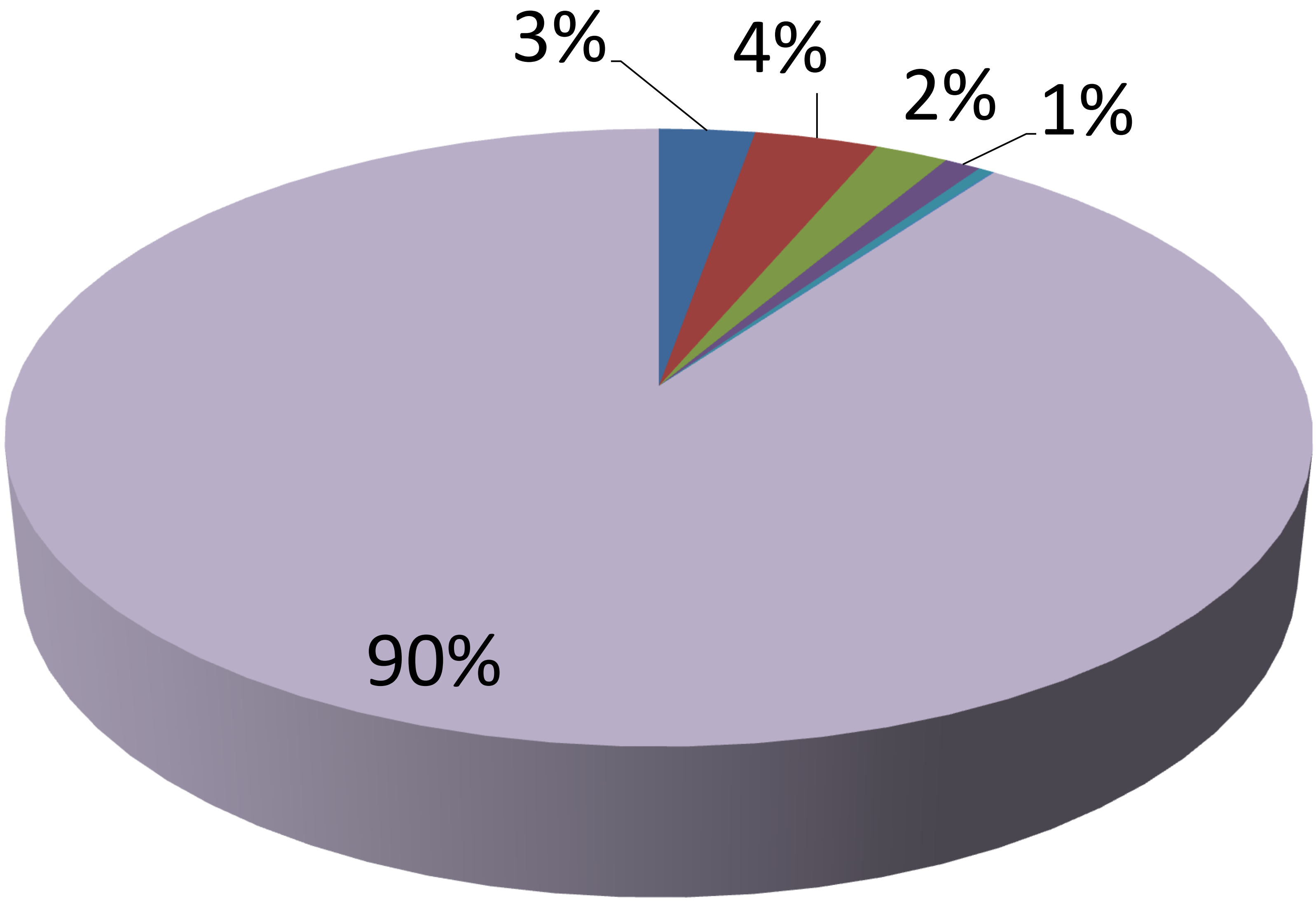

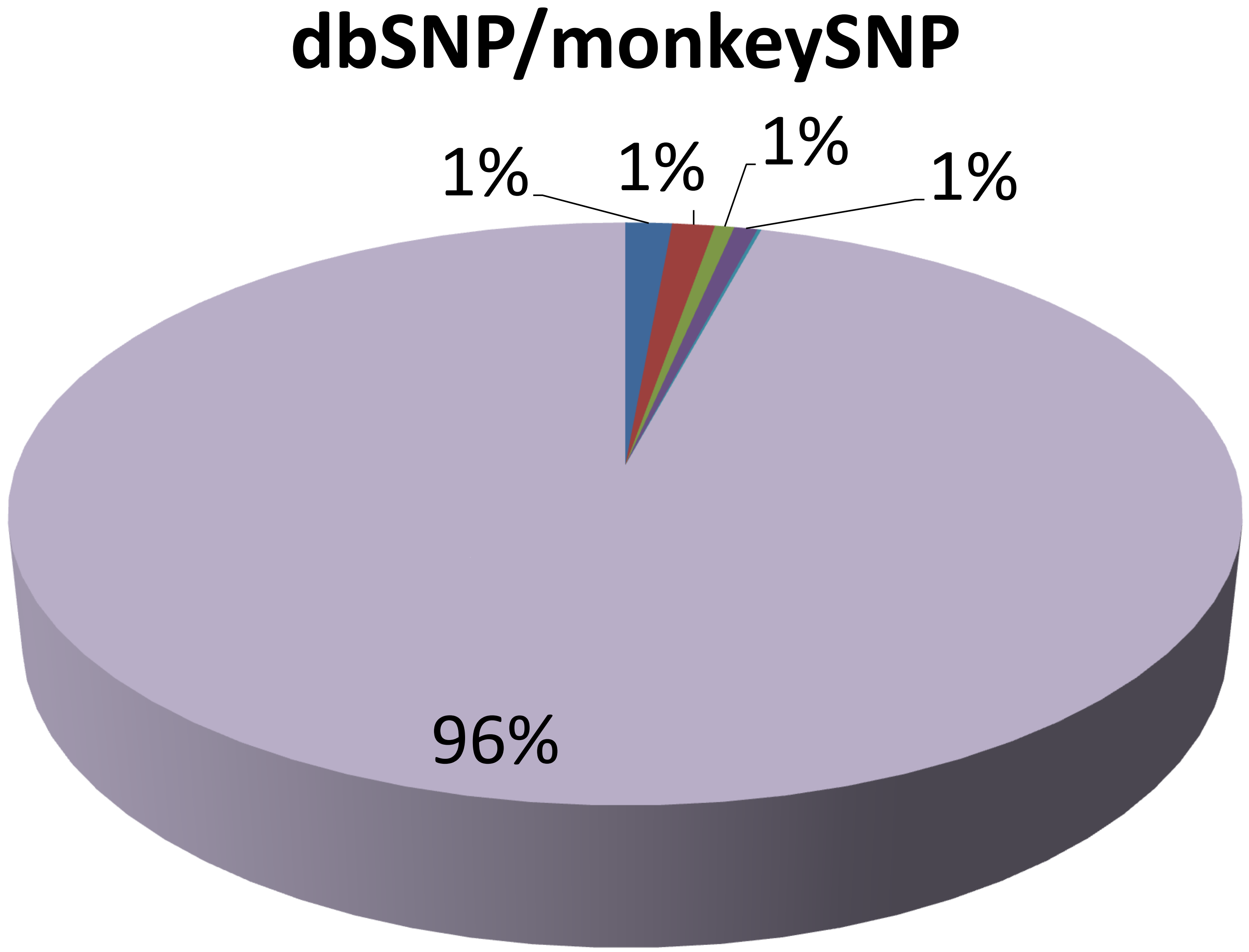
dbSNP/monkeySNP
1%
1%
1%
1%
96%

egeno.assembly.het

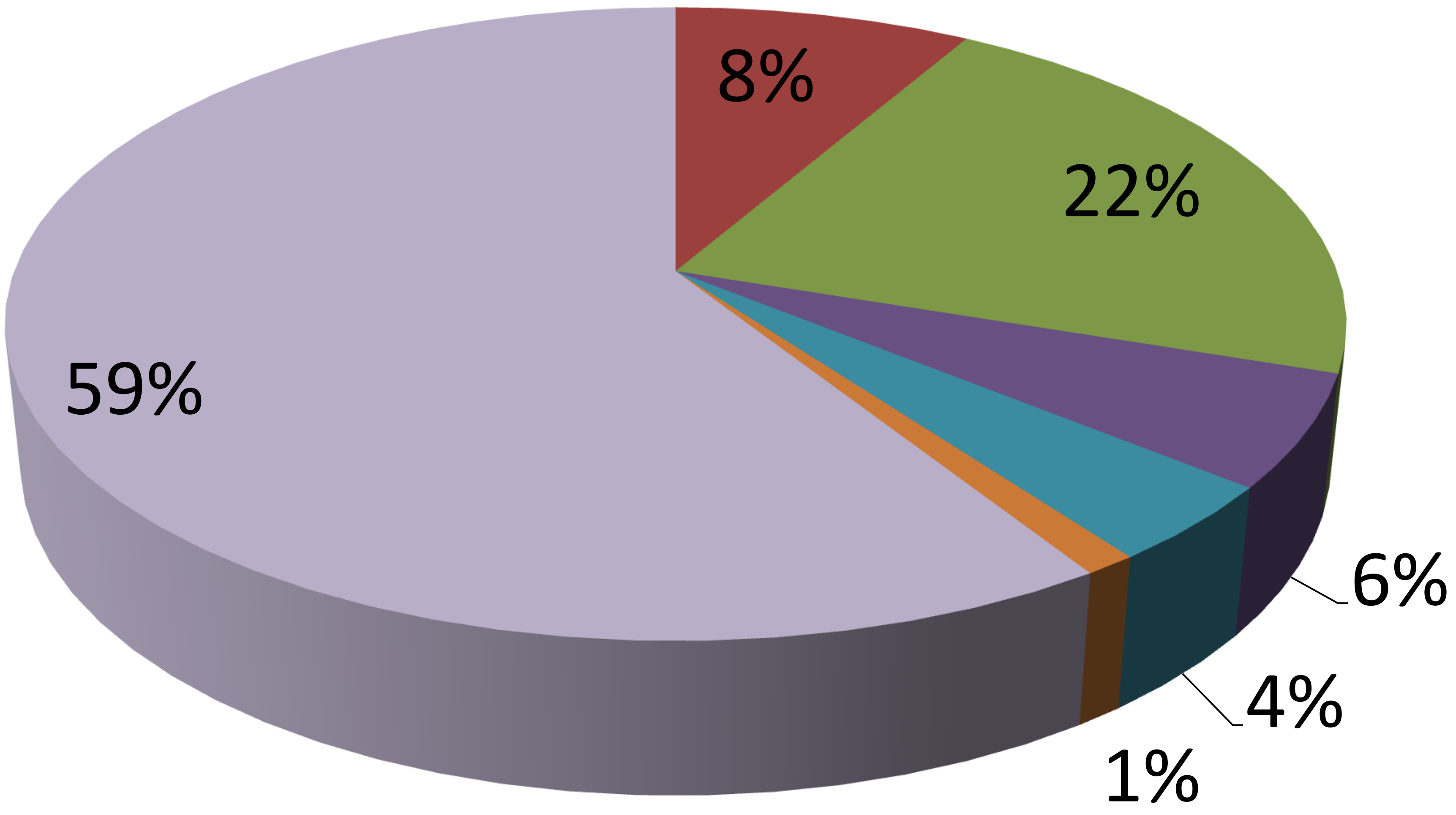

egeno.17573.frag

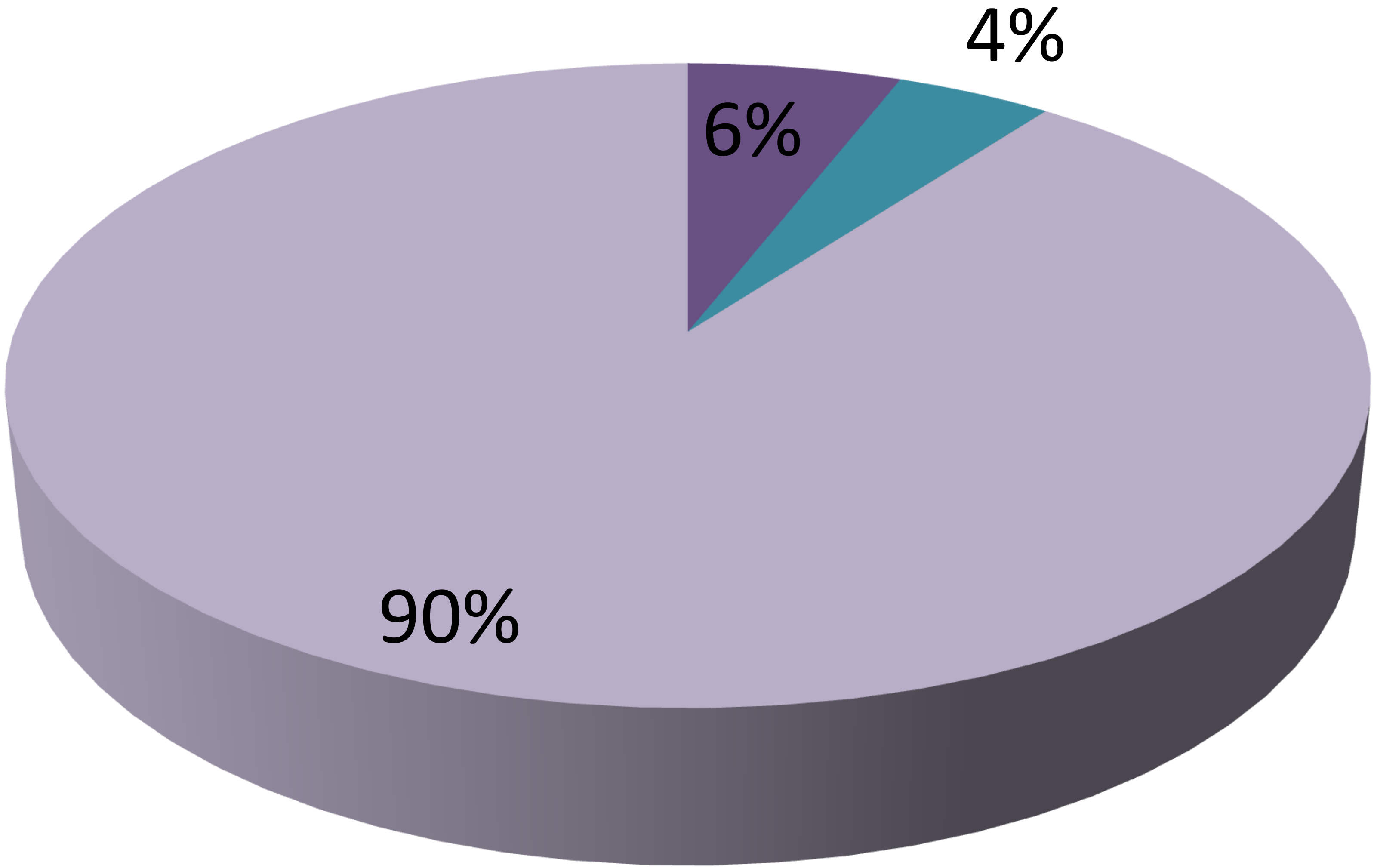

egeno.17573.mp

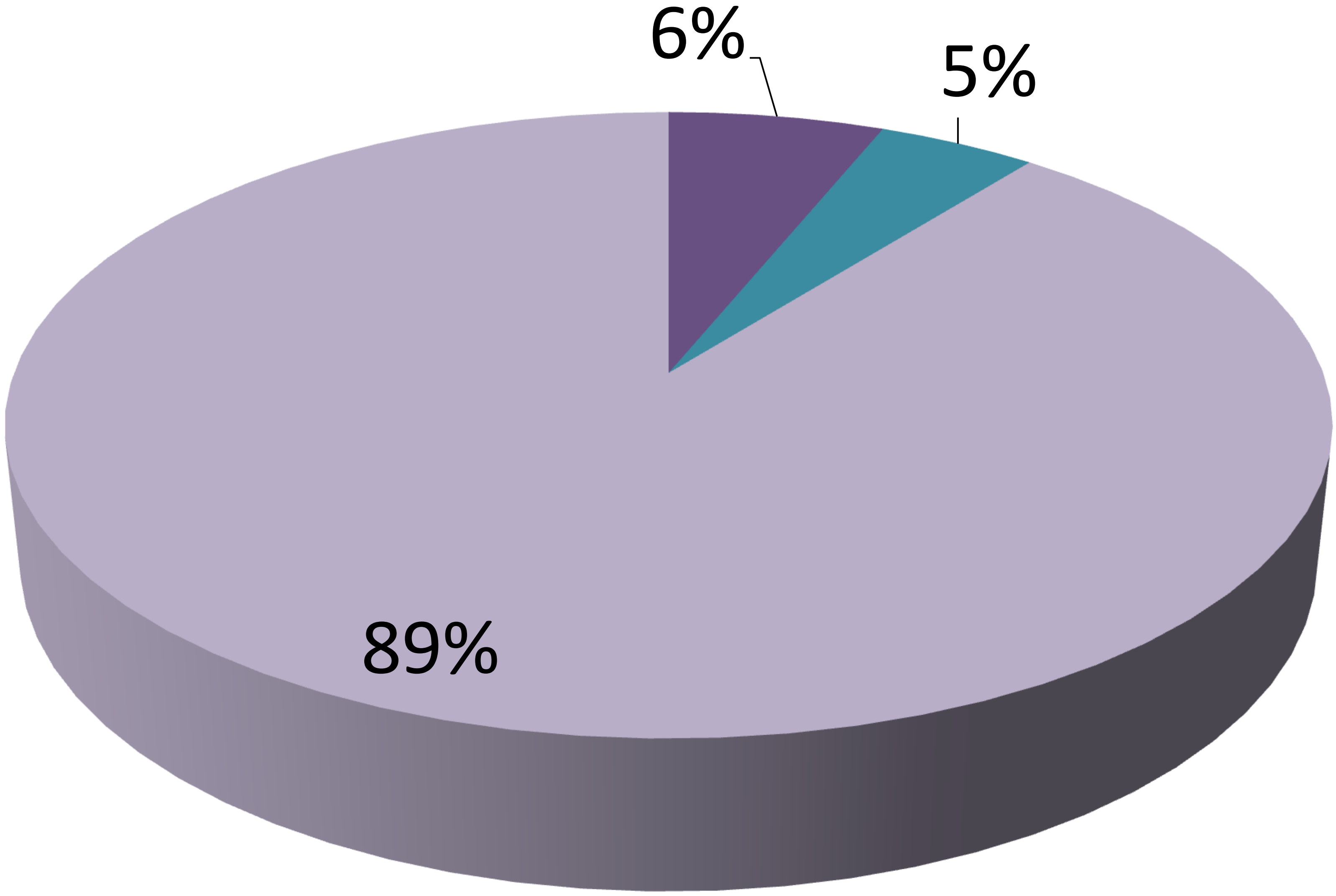

egeno.r1766.frag

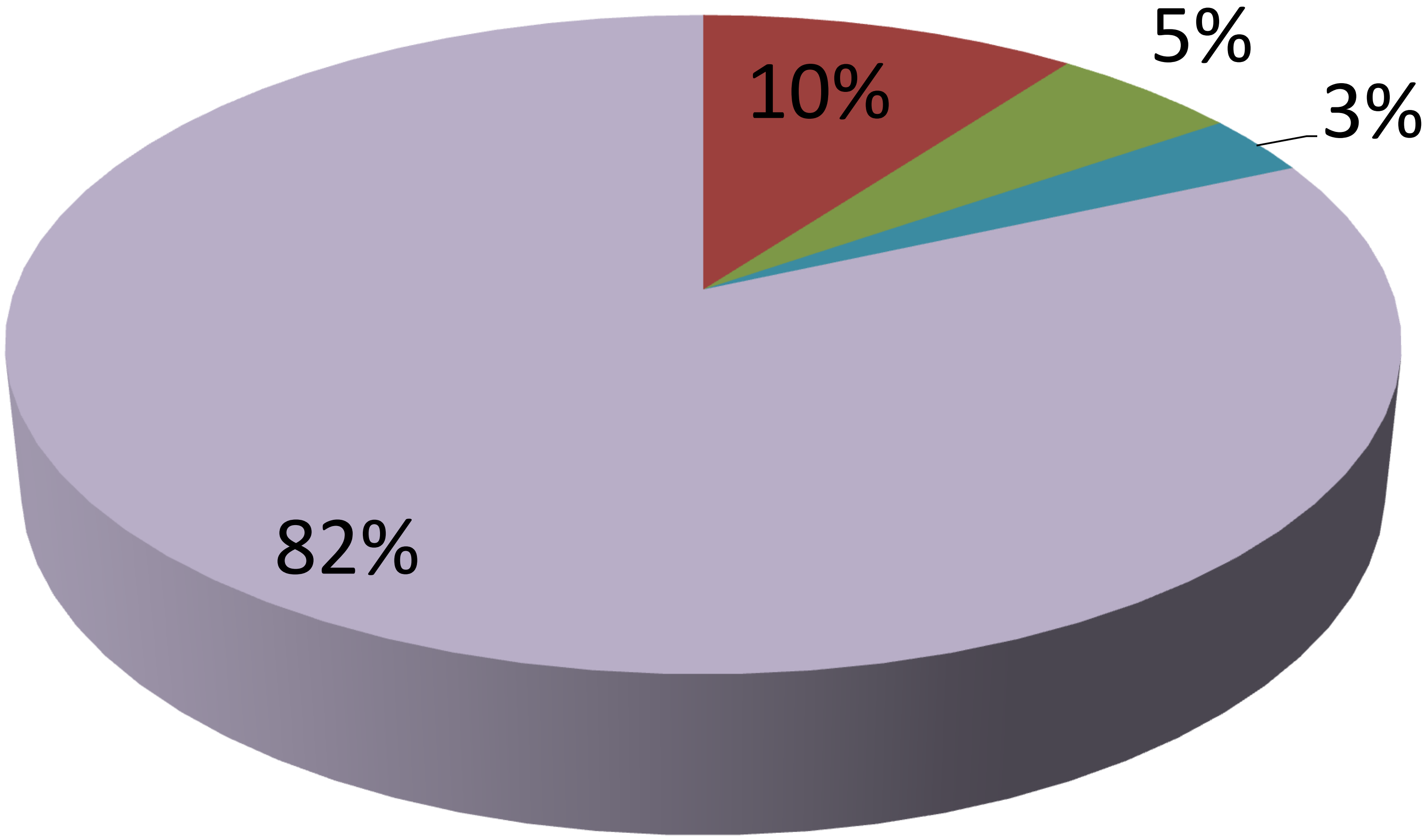

egeno.r02120.frag

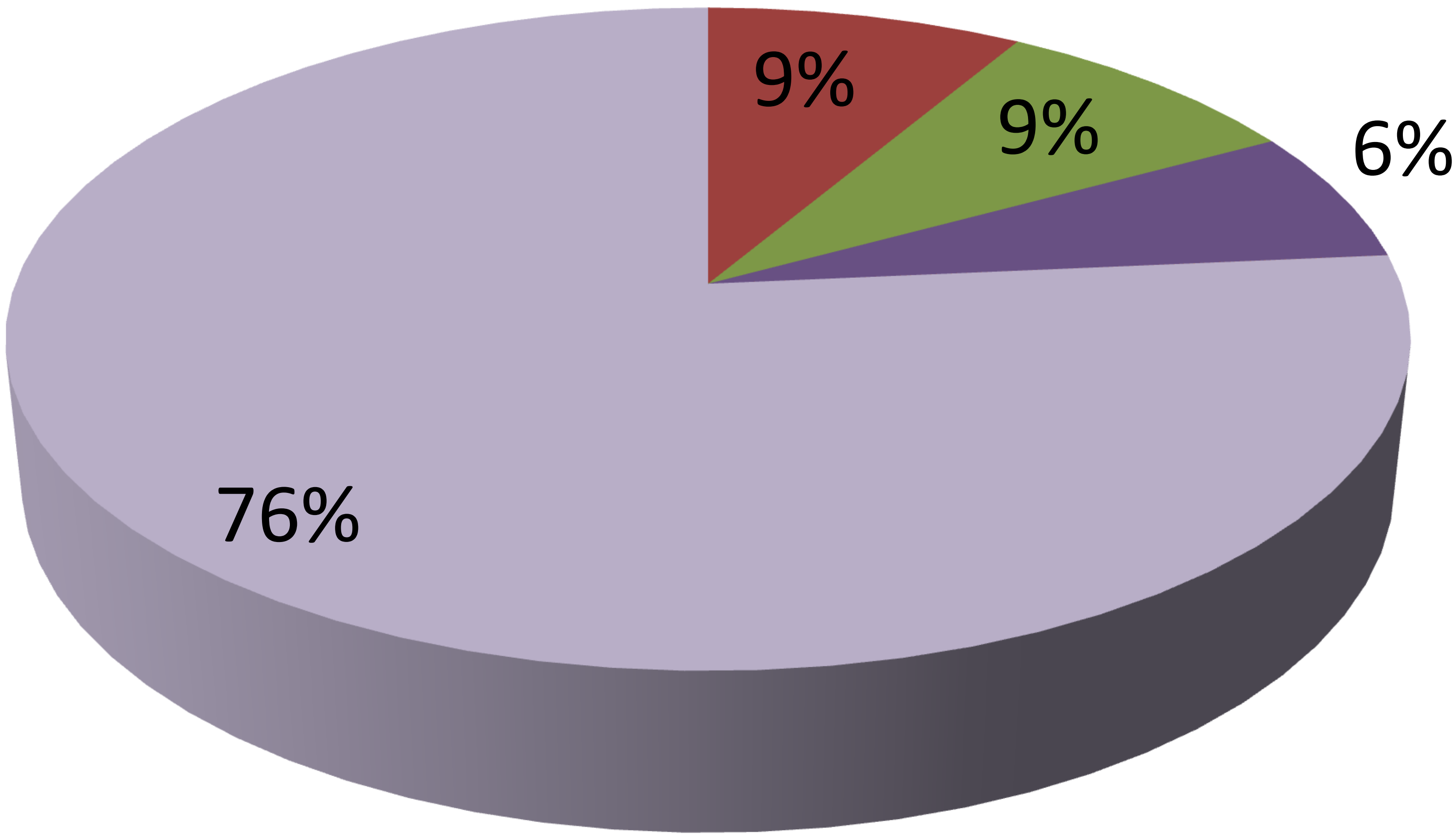

egeno.sub-species comp

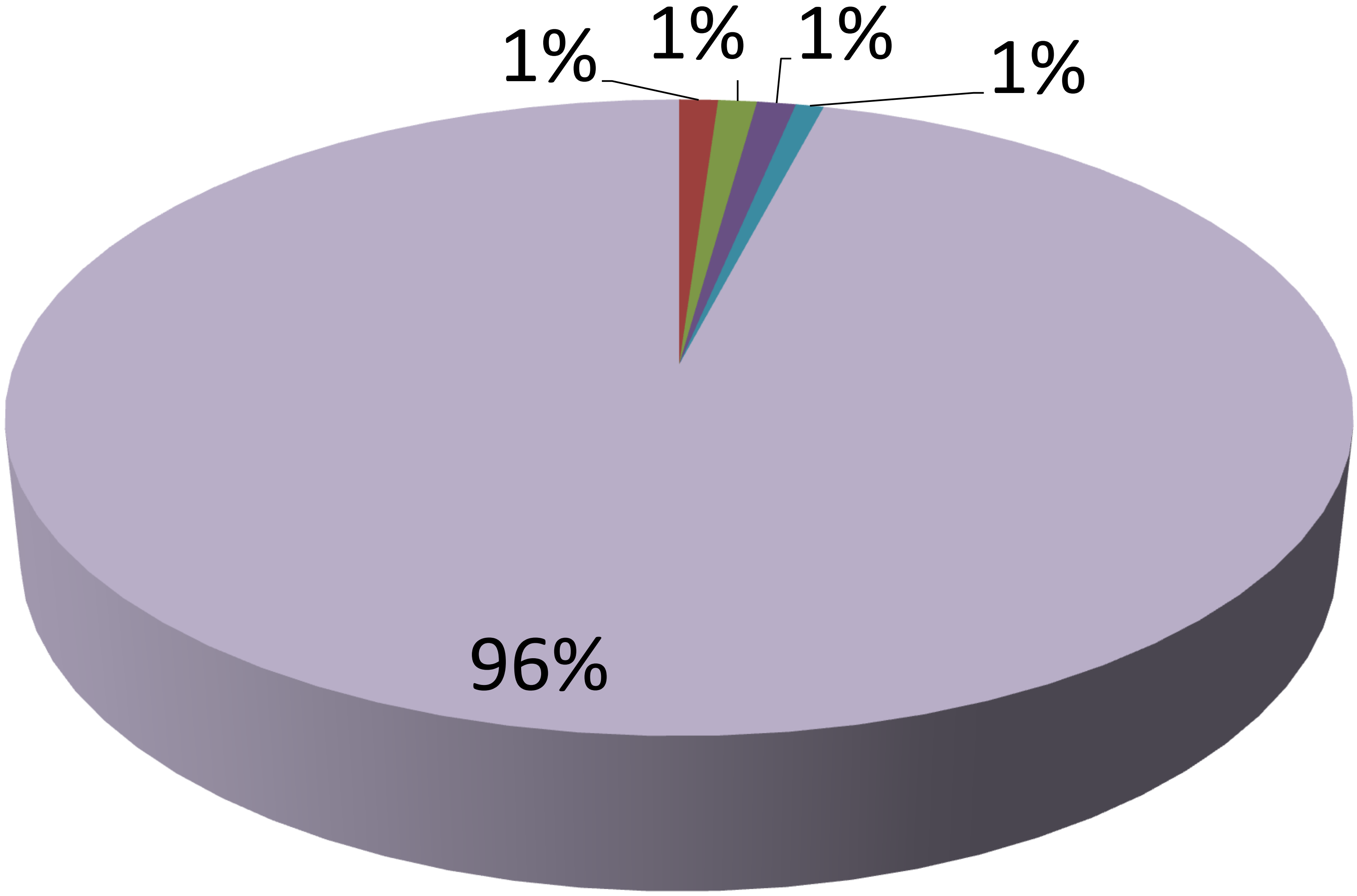

egeno.sub-species comp

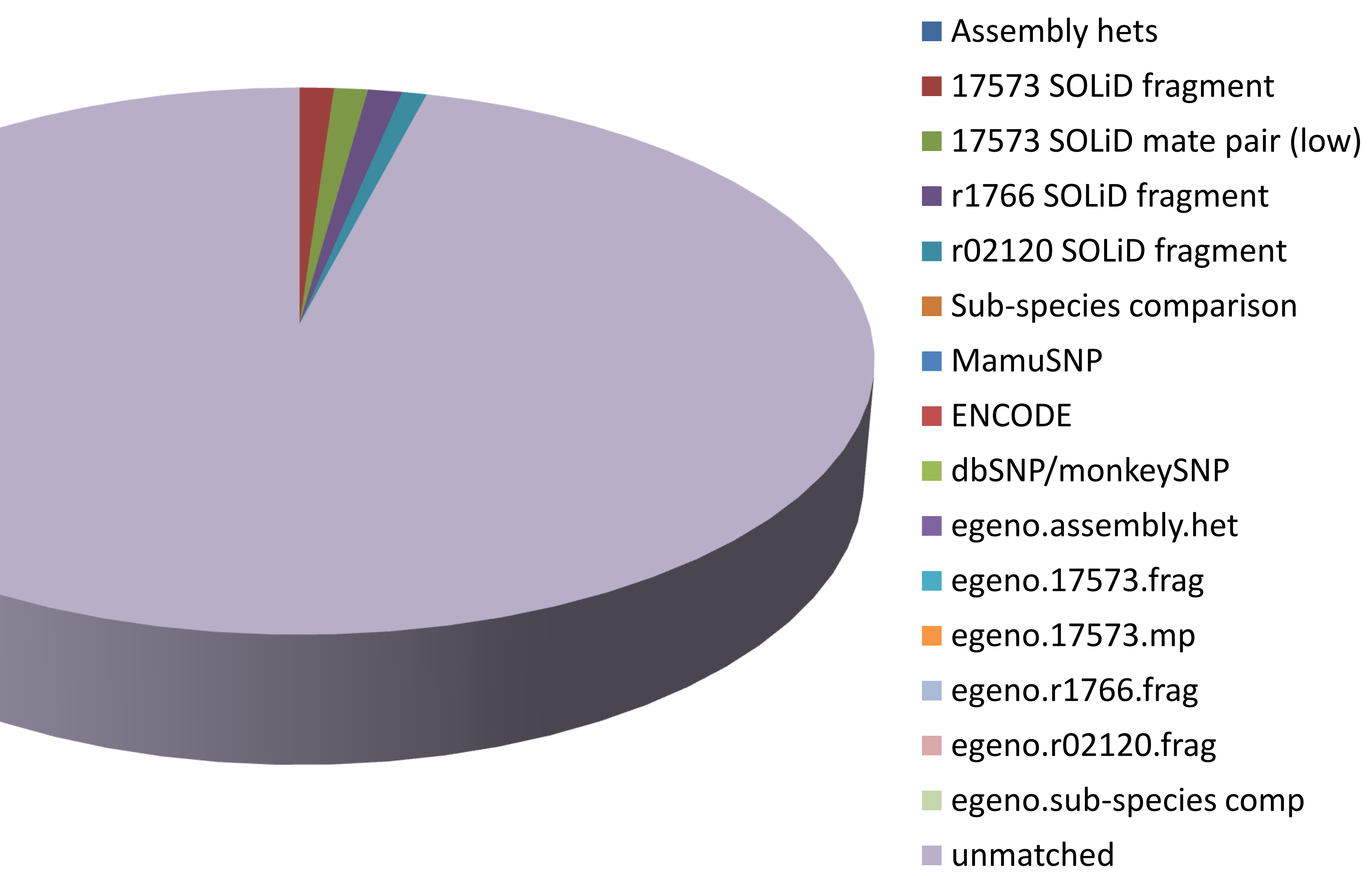

Assembly hets
17573 SOLiD fragment
17573 SOLiD mate pair (low)
r1766 SOLiD fragment
r02120 SOLiD fragment
Sub-species comparison
MamuSNP
ENCODE
dbSNP/monkeySNP
egeno.assembly.het
egeno.17573.frag
egeno.17573.mp
egeno.r1766.frag
egeno.r02120.frag
egeno.sub-species comp
unmatched
