# Supplementary figures and images for: Characterization of single-nucleotide variation in Indian-origin rhesus macaques (Macaca mulatta)
Source: BMC Genomics. 2011 Jun 13;12:311. doi: 10.1186/1471-2164-12-311 (PMC3141668; doi:10.1186/1471-2164-12-311)

# Read coverage histogram

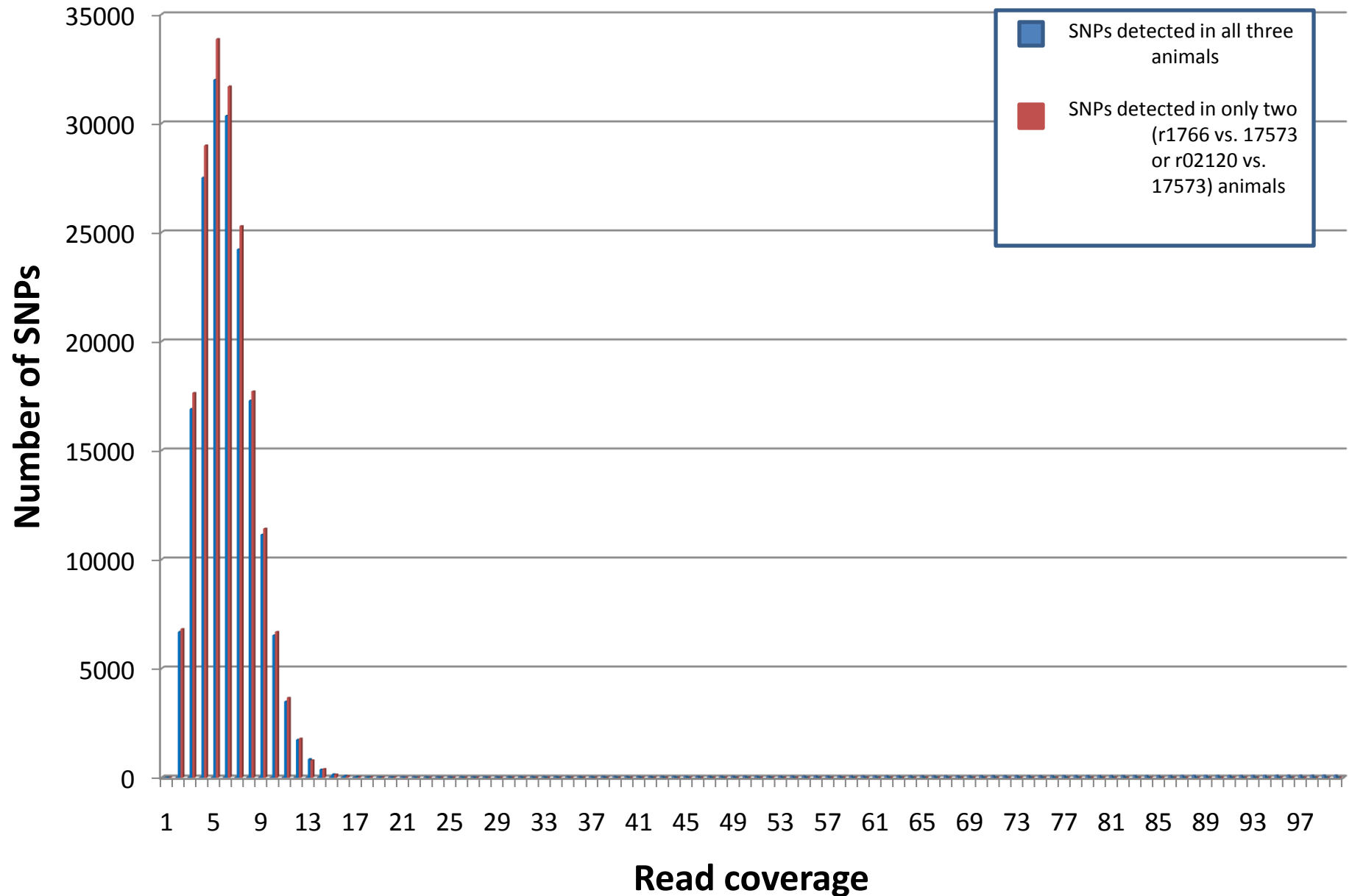

Supplement: Additional file 2 — Figure S2, Additional file 2. Sanger read coverage for validated SNPs. All SNPs that were found in common between r1766, r02120, and 17573 (306,782, Figure 1B) were tested for Sanger read coverage using the 17573 Sanger data by looking for the same location and allele SNP call in the Sanger output (red bars). Average genome-wide Sanger read coverage was 5.2X. We analyzed an identical number of randomly selected SNPs that were detected in only two of the three resequenced animals (blue bars) to test if there were differences in the distribution of read coverage for the two SNP data sets. The distributions are indistinguishable, however, and the proportions of tested SNPs exhibiting 11+ read coverage are not statistically different (3.8% for SNPs in all three animals, 3.6% for SNPs detected in only two animals). [file 1471-2164-12-311-S2.PDF]

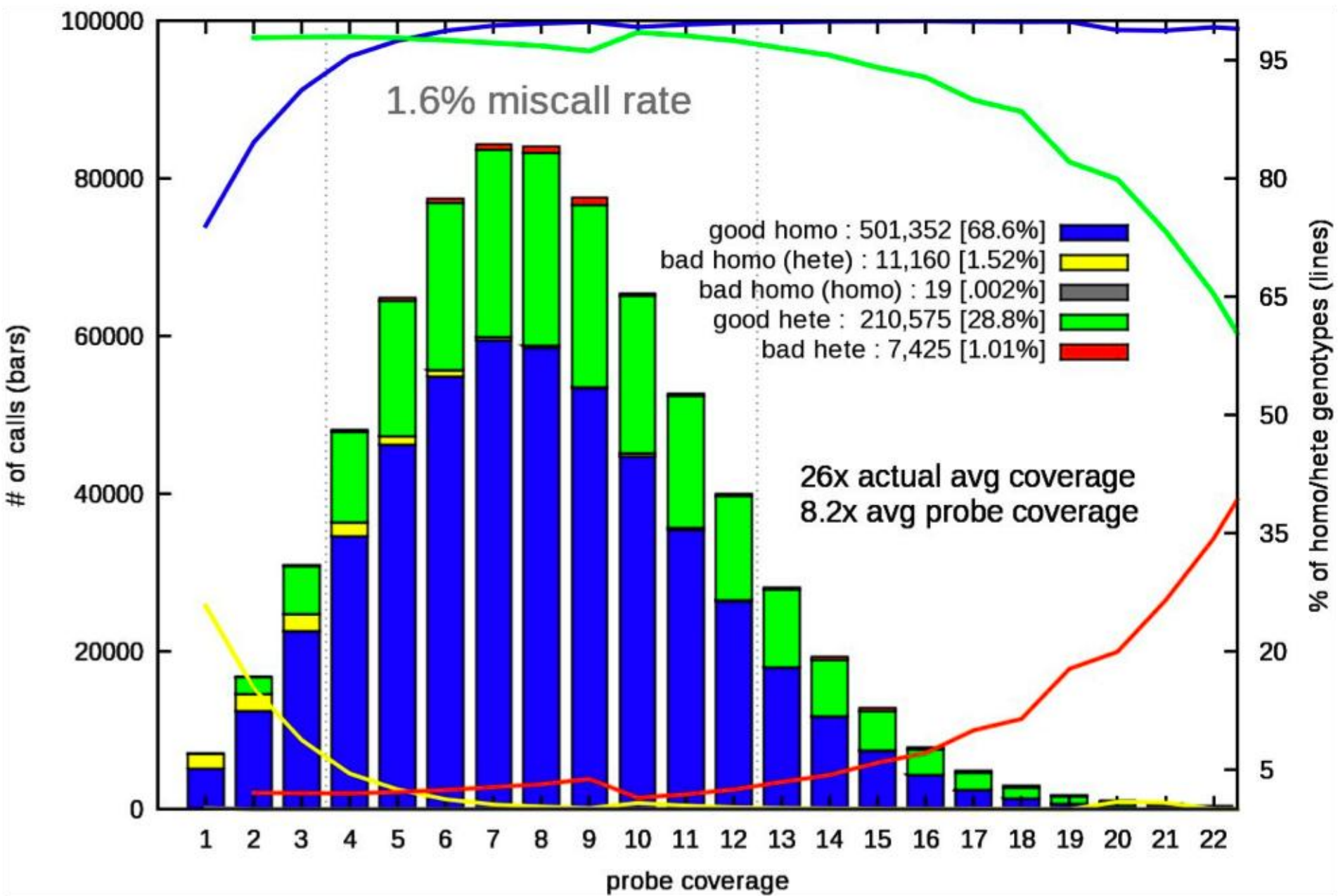

Supplement: Additional file 4 — Figure S3, Additional file 4. dbSNP concordance of e-genotype. One human sample of SOLiD data (26X coverage) from the 1000 Genomes pilot data was analyzed for the ~1 million SNPs identified in the project for that sample. Color coding for the bars as well as the lines is as follows: good homozygous calls (blue), heterozygotes called as homozygotes (yellow), homozygotes called as the wrong homozygote (grey), good heterozygote calls (green), and erroneously called heterozygotes (red). The total miscall rate was ~2.6%. Excluding very high and very low coverage errors (shown outside of dotted lines, due to bad heterozygous SNP calls from repetitive regions or coverage too low to detect heterozygotes, respectively) the miscall rate was determined to be 1.6%. Overall probe coverage was reduced to 8.2X due to the extremely stringent requirement of exact matching of probes for the full 31 bp probe length. Percentages of homozygous or heterozygous calls that fit into each category of good or error calls were linearly graphed relative to probe coverage, indicating that errors were much more likely at very high and very low probe coverage, while good calls were most likely in intermediate coverage ranges. [file 1471-2164-12-311-S4.PDF]
